# The uptake characteristics of Prussian-blue nanoparticles for rare metal ions for recycling of precious metals from nuclear and electronic wastes

**DOI:** 10.1038/s41598-022-08838-1

**Published:** 2022-03-24

**Authors:** Shinta Watanabe, Yusuke Inaba, Miki Harigai, Kenji Takeshita, Jun Onoe

**Affiliations:** 1 Department of Energy Science and Engineering, Nagoya University, Furo-cho, Chikusa-ku, Nagoya, Aichi 464-8603 Japan; 2 Laboratory for Zero-Carbon Energy, Tokyo Institute of Technology, 2-12-1-N1-16, O-okayama, Meguro-ku, Tokyo, 152-8550 Japan

**Keywords:** Environmental sciences, Nanoscience and technology

## Abstract

We have examined the uptake mechanisms of platinum-group-metals (PGMs) and molybdenum (Mo) ions into Prussian blue nanoparticles (PBNPs) in a nitric acid solution for 24-h sorption test, using inductively coupled plasma atomic emission spectroscopy, powder XRD, and UV–Vis-NIR spectroscopy in combination with first-principles calculations, and revealed that the Ru^4+^ and Pd^2+^ ions are incorporated into PBNPs by substitution with Fe^3+^ and Fe^2+^ ions of the PB framework, respectively, whereas the Rh^3+^ ion is incorporated into PBNPs by substitution mainly with Fe^3+^ and minorly with Fe^2+^ ion, and Mo^6+^ ion is incorporated into PBNPs by substitution with both Fe^2+^ and Fe^3+^ ions, with maintaining the crystal structure before and after the sorption test. Assuming that the amount of Fe elusion is equal to that of PGMs/Mo substitution, the substitution efficiency is estimated to be 39.0% for Ru, 47.8% for Rh, 87% for Pd, and 17.1% for Mo^6+^. This implies that 0.13 g of Ru, 0.16 g of Rh, 0.30 g of Pd, and 0.107 g of Mo can be recovered by using 1 g PBNPs with a chemical form of KFe(III)[Fe(II)(CN)_6_].

## Introduction

Recovery of precious metals from both nuclear wastes (N-wastes) and electronic wastes (E-wastes) plays one of key roles in solving energy and environmental issues in order to maintain our sustainable developing society. For the former one (N-wastes), the spent nuclear fuel generated from the power plants are vitrified at the reprocessing plant. After separating uranium (U) and plutonium (Pu) from the spent fuels by using the PUREX (**P**lutonium **U**ranium **R**edox **EX**traction) method in order to re-use them as new fuels, the high-level radioactive liquid wastes (HLLW) are vitrified and geologically disposed^1–3^. In the vitrification processes, platinum-group metals (PGMs: especially, ruthenium: Ru, rhodium: Rh, and palladium: Pd) and molybdenum (Mo) cause serious problems: (1) PGMs tend to settle on the sidewall surface of a glass melter, giving rise to an inhomogeneous thermal distribution of the melter, and (2) Mo forms low-viscosity fluid compounds so-called “yellow phase” in the vitrified object^4,5^. These issues degrade the quality and stability of the vitrified objects due to heterogeneity, and increase both disposal spaces and costs in conjunction with additional vitrified rods produced by flushing the glass melter. Considering that Ru, Rh, and Pd with an amount of 2.09, 0.36, and 1.20 kg, respectively, are generated in 1 t of used nuclear fuels (burnup: 30,000 MWd/t, cooling period: 150 days) for light-water reactors (those generated amounts will increase by 1.5–2 times for fast breeder reactors), it is useful to recover PGMs from HLLW not only for the disposal of N-wastes but also for the recycling of precious metals from our alternative perspective. It is, of course, noted that it takes few decades to reduce their radioactive levels below safety standard except ^107^Pd long-lifetime nuclide (half-life: 6.5 million years) that is planned to be deleted using an extinction process with fast neutrons. In a similar manner to PGMs, Mo is also used both as Mo-Cu alloys and MoS_2_ materials for electronic and car industries, respectively, and also used as an alternative material for tungsten (W) because of its lower price.

For the latter case (E-wastes), the abundance of precious metals (Ru, Rh, Pd, rhenium: Re, osmium: Os, iridium: Ir, platinum: Pt, gold: Au) is much smaller by 10^–6^–10^–9^ times than that of rock-forming elements (sodium: Na, magnesium: Mg, aluminum: Al, silicon: Si, potassium: K, calcium: Ca, and iron: Fe)^6^. Although the precious elements are not uniformly dotted over the world in nature, the much amount of them has, however, been stored in E-wastes so far. For example, the amount of Au contained in 1 t of mobile phones is 300–400 g, which is much higher by 10–80 times than that in 1 t of natural ore. The other elements have a similar situation to Au. Consequently, the recovery of those precious elements from E-wastes is much more effective and efficient when compared to their collections from natural ore.

The aim of the present study is to solve the environmental and energy issues by utilizing the nanospace of Prussian blue (PB), which is one of metal hexacyanoferrates (MHCFs), as a sorbent^7,8^. Since MHCFs have a simple cubic lattice structure like a jungle gym (inset in Fig. 2), in which divalent (M^2+^) and trivalent (M^3+^) metal cations are cross-linked with each other via cyano-group anion (CN^–^), and exhibit many fascinating features^9–11^, they have been extensively investigated from viewpoints of both scientific and industrial aspects^12–18^. Recently, PB (FeHCF) has been applied to remove radioactive cesium-134 (^134^Cs) and ^137^Cs elements from contaminated soils caused by Fukushima nuclear plant accident in 2011^19–23^, because PB has the jungle-gym cubic structure with a 0.5 nm-interstitial site (4*c* site in $F\overline{4} 3m$ space group) that plays a role in trapping Cs ions efficiently. However, the uptake mechanisms of PB for multi-valent metal ions have been still unclear so far, though three possible sorption processes can be considered: (1) surface adsorption, (2) insertion or diffusion into interstitial sites (nanospace) in a similar manner to Cs ions, and (3) substitution of Fe with metals in the framework of PBs. In order to develop high-performance MHCF sorbents for recovery of the precious metals from N- and E-wastes, it is necessary to unravel the uptake characteristics of PB for those metal ions.

More recently, we have investigated the uptake mechanism of Pd (one of the most important elements in industry such as catalyst) ion into PB nanoparticles (PBNPs) in a nitric acid solution, and revealed that the Pd^2+^ ion is incorporated into PBNPs by substitution with Fe^2+^ ion of the PB framework with maintaining the crystal structure before and after Pd sorption, and the substitution efficiency was estimated to be 87% per PB unit cell^24^. This implies that 0.30 g Pd can be recovered by using 1 g PB with a chemical form of KFe(III)[Fe(II)(CN)_6_].

In the present study, we report here on the uptake characteristics of PBNPs for PGMs/Mo ions in a nitric acid solution as well as Pd ion. The uptake efficiency of PGMs/Mo into PBNPs and the elution efficiency of Fe from PBNPs were measured using inductively coupled plasma atomic emission spectroscopy (ICP-AES) before and after 24 h sorption test. We also examined changes in the structural and electronic properties of PBNPs before and after the sorption test, using powder x-ray diffraction (XRD) and ultraviolet–visible-Near IR (UV–vis-NIR) spectroscopy, in combination with first-principles calculations based on density functional theory. Furthermore, in order to understand the difference in the sorption efficiency among PGMs/Mo ions, we estimated the surface adsorption, diffusion, and substitution energies when PGMs/Mo ions are incorporated into PB unit cell, using the first-principles calculations.

## Experimental and theoretical methods

### Experimental methods

PBNPs were synthesized by mixing potassium hexacyanoferrate (II) (5 mmol, K_4_[Fe(CN)_6_]·3H_2_O, KANTO CHEMICAL) with Fe(III) nitrate (10 mmol, Fe(NO_3_)·9H_2_O, WAKO) in aqueous solution. PB precipitates thus formed were rinsed with ultrapure water after centrifugation (3000 rpm), which was performed for five times. Thereafter, PBNPs were dried at 75 °C for 12 h and thereafter kept in the vacuum desiccator for 3 h. We already confirmed the structural and physicochemical characteristics of PBNPs used in the present work elsewhere^24^.

Sorption test of PGMs/Mo ion (1 mM) for PBNPs (500 mg) were carried out in 1.5 M nitric acid solution (10 mL) upon shaking for 24 h. Subsequently, the mixtures were centrifuged to separate the PBNPs from the solution, and the concentration (*C*) of PGMs/Mo ion in the supernatant liquid was measured before (*C*_initial_) and after (*C*_final_) the test, using ICP-AES (ICPE-9000, SHIMADZU), in order to estimate the sorption efficiency, [(*C*_initial_ − *C*_final_)/*C*_initial_ × 100%], of PGMs/Mo ions into PBNPs. We also measured the concentration of Fe ion in the supernatant liquid after the sorption test, and estimated the elution efficiency of Fe ion when compared to the initial amount of PBNPs. Details of sorption test conditions have been described elsewhere^25^.

Powder XRD patterns and UV–vis-NIR diffuse reflectance spectra of the pristine and PGMs/Mo-sorbed PBNPs were measured using Rigaku RINT2200 (Cu Kα) and Shimazu UV-2600 spectrometer, respectively. The diffuse reflectance spectra thus obtained were converted to the corresponding absorption spectra in terms of the Kubelka–Munk conversion equation.

### Theoretical methods

**Figure 1:**
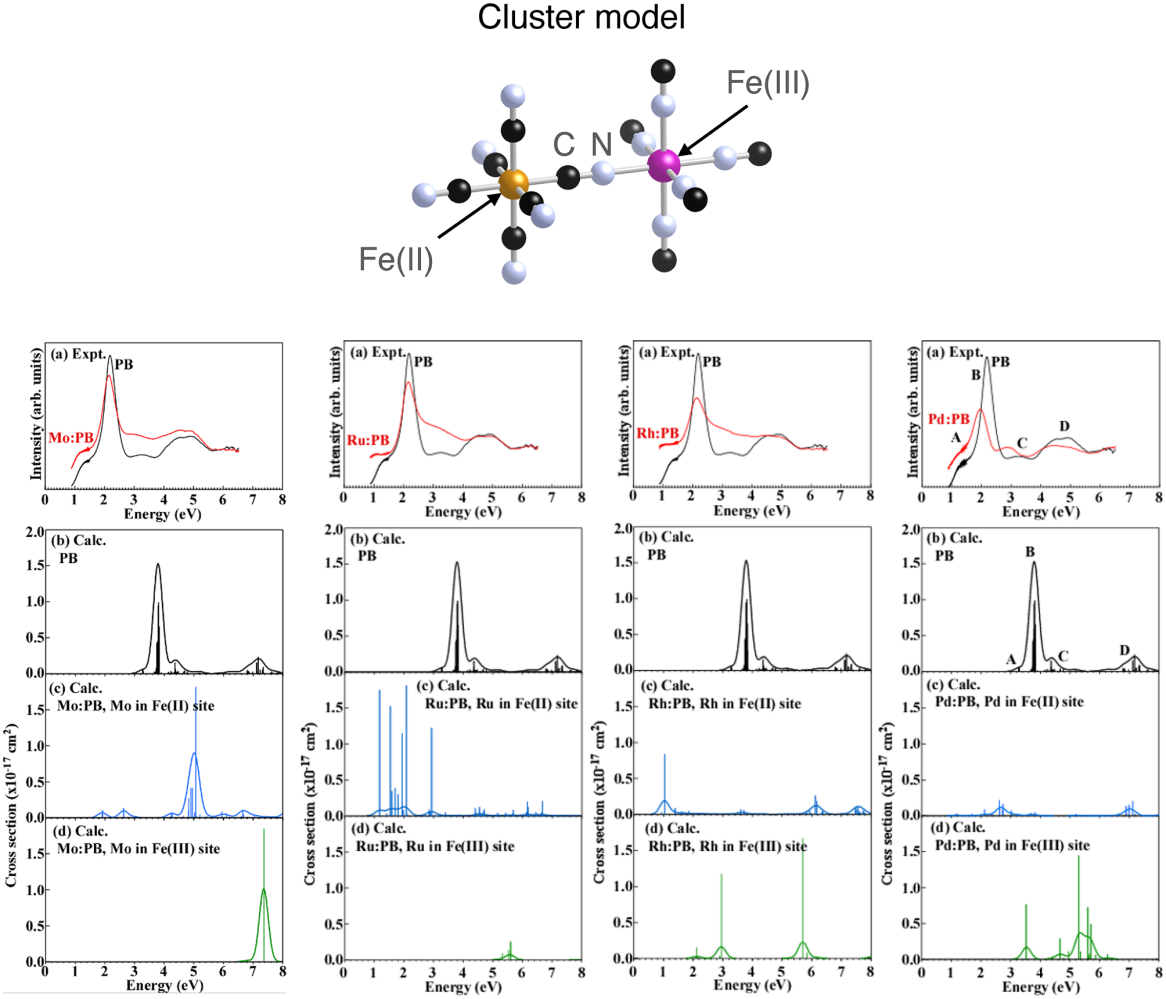
Theoretical spectra of the pristine (**b**) and PGMs/Mo-sorbed PB using the cluster models for Fe^2+^ (**c**) and Fe^3+^ (**d**) substitution, along with the experimental UV–vis-NIR spectra of PB before (black) and after (red) PGMs sorption (**a**).

Theoretical absorption spectra of the pristine and PGMs/Mo-sorbed PBs were obtained using the relativistic configuration interaction (CI) method^26,27^, because the present method has been already confirmed to reproduce the experimental absorption spectra of Fe, Co, and Ni ferrocyanides quantitatively, using Fe(II)M(III)(CN^–^)_11_^6–^ (M = Fe, Co, Ni) cluster model^28^. 0 The multiplet energy levels and absorption spectra were calculated using Fe^2+^Fe^3+^(CN^–^)_11_^6–^, MFe^3+^(CN^–^)_11_^6–^, and Fe^2+^M(CN^–^)_11_^6–^ cluster models (M = Mo^6+^, Ru^4+^, Rh^3+^, Pd^2+^) (see Fig. 1), respectively, for the pristine and PGMs/Mo-sorbed PBNPs. Here, when the PBNPs are applied to the disposal of HLLW in a strong nitric acid solution (2–8 M), the present calculations were performed using an oxidation ionic valence of 6+, 4+, 3+, and 2+ for Mo, Ru, Rh, and Pd, respectively. The choice of the valence values for these ions is reasonable, because the valence of each ion in the nitric acid solution has been already clarified experimentally^29–33^. The structural parameters of these cluster models were determined from the optimized structures obtained using density functional theory (DFT) calculations. Details of the CI calculation method were described elsewhere^26,27^. As the active space for the CI calculations, all the electronic configurations of the d–d transitions for Fe and PGMs/Mo ions were explicitly treated. On the other hand, the electronic configurations for the charge transfer (CT) transitions from Fe^2+^/M to M were considered up to two electron excitations. The oscillator strength of the electric dipole transitions averaged over all directions was obtained using the general equation expressing the electric dipole transition. Theoretical absorption spectra were obtained by convolution of the oscillator strength replaced with a 0.30 eV full-width-at-half-maximum (FWHM) Gaussian function.

The surface adsorption, diffusion, and substitution energies of PGMs/Mo ions when incorporated into PBNPs were estimated using the CASTEP^34^ and QUANTUM-ESPRESSO^35,36^ based on DFT^37,38^. We adopted the Vanderbilt-type ultrasoft pseudopotentials^39^ throughout all the present calculations. The exchange–correlation potential was considered using the GGA (PBE)^40^. The cut-off energy of the plane wave was 550 eV, and the Brillouin zone was sampled on the 2 × 2 × 2 and 4 × 4 × 1 Monkhorst–Pack grid^41^ for the unit cell and (100) surface models of PB, respectively.

The adsorption energy of PGMs/Mo ions on the (100) surface of PB were calculated using a Fe(III)_8_Fe(II)_8_(CN)_40_ model (see Fig. 3) consisting of two layers of (100) surface (see Fig. 3a side view). In order to reproduce the surface environment, the 10 Å vacuum region was set on the (100) surface of PB model. The surface adsorption energy (*E*_ad_) was evaluated using the following equation,

1 $$ E_{{{\text{ad}}}} = E_{{\text{T}}} \left[ {\text{PGMs/Mo:PB}} \right]{-}\left( {E_{{\text{T}}} \left[ {{\text{PB}}} \right] + E_{{\text{T}}} \left[ {\text{PGMs/Mo}} \right]} \right). $$

Here, each term denotes the total energy of PGM/Mo-sorbed PBs, pristine PB, and PGMs/Mo themselves, respectively, which were calculated using CASTEP. The maximally-localized Wannier functions (MLWFs) of the C2p and N2p atomic orbitals (AOs) on the (100) surface of PB were obtained using Wannier90^42^.

The diffusion energy of PGMs/Mo ions through the nanospace of PBNPs was estimated for the unit cell model by using the Nudged elastic band (NEB) method implemented in QUANTUM-ESPRESSO. A diffusion pathway for PGMs/Mo ions in the nanospace of PBNPs was considered to be a route from the center of the square made of the four Fe ions in the (100) plane to the same site of the adjacent (100) plane via the 4*c* site (see the inset of Fig. 4).

The substitution energy (*E*_S_) of PGMs/Mo with Fe of PB was estimated for the unit cell model consisting of 60 atoms, which corresponds to 12.5% Fe ions substituted with PGMs/Mo ions. The *E*_S_ was evaluated using the following equation,

2 $$ E_{{\text{S}}} = E_{{\text{T}}} \left[ {\text{PGMs/Mo:PB}} \right]{-}E_{{\text{T}}} \left[ {{\text{PB}}} \right] + \mu_{{{\text{Fe}}}} {-}\mu_{{\text{PGMs/Mo}}} $$

Here, the first and second terms denote the total energy of the PGMs/Mo-sorbed and pristine PB models, respectively, whereas the third and fourth terms denote the chemical potentials of Fe and PGMs/Mo, respectively. In the present study, we used the chemical potential of the oxide at the oxidation limit and of the neutral atom at the reduction limit. These calculations were performed by DFT + *U* method^43^, using CASTEP. The *U* value was set to be 7.0 eV for the high-spin (HS) state of Fe^3+^ (Ionic radius: 0.645 Å for six coordination number), 3.0 eV for the low-spin (LS) state of Fe^2+^ (Ionic radius: 0.61 Å for six coordination number), and 2.0 eV for the LS state of PGMs/Mo (Ionic radius: 0.59 Å for Mo^6+^ with six coordination number, 0.62 Å for Ru^4+^ with six coordination number, 0.665 Å for Rh^3+^ with six coordination number, and 0.86 Å for Pa^2+^ with six coordination number)^44–46^. All calculations in the present study were performed until the residual forces and stresses below 0.01 eV/Å and 0.02 GPa, respectively. A uniformed background charge, so called the Jellium model, was used to accommodate the non-neutral states.

## Results and discussion

Table 1 summarizes the sorption efficiency of PGM/Mo ions, the elution efficiency of Fe ion from PBNPs, the substitution efficiency of PGMs/Mo with Fe when incorporated into PBNPs, and the estimated amount of PGM/Mo recovery per 1 g PBNPs after 24 h sorption test. Since we already unraveled the uptake mechanism for Pd ion exhibiting the highest sorption and elution efficiencies^24^, we mainly discuss the mechanism for Ru, Rh, and Mo ions in relation to the results of Table 1.

**Table 1:** The sorption efficiency of PGMs/Mo ions into PBNPs, the elution efficiency of Fe ion from PBNPs after 24 h sorption test, the substitution efficiency of PGMs/Mo with Fe^2+^ or Fe^3+^ ion, the substitution site, and the amount of recovery per 1 g PB.

	Ru	Rh	Pd	Mo
Sorption efficiency (%)	33.1	68.2	99.9	51.7
Elution efficiency of Fe ion (%)	19.5	23.9	43.5	17.1
Substitution efficiency (%)	39.0	47.8	87.0	17.1
Substitution site	Fe^3+^	Fe^3+^	Fe^2+^	Fe^2+^/Fe^3+^
Amount of recovery per 1 g PB* (g)	0.128	0.160	0.302	0.107

*Chemical form: KFe(III)[Fe(II)(CN)6].

Sorption efficiency [%] = [(*C*initial − *C*final)/*C*initial] × 100.

Here, *C*initial and *C*final denote the concentration of Pd ion in nitric acid solution before and after 24-h sorption test, respectively.

### UV–vis-NIR spectra

As shown in Table 1, the elution efficiency of Fe ion from PBNPs strongly supports that Ru, Rh, and Mo ions are also substituted with Fe ion of PBNPs when incorporated into PBNPs as well as Pd ion. In order to confirm the substitution of PGMs/Mo ions with Fe ion, we examined the UV–vis-NIR spectra of PBNPs before and after PGMs/Mo sorption in combination with first-principles calculations of theoretical spectra for the simple PB cluster model (see Fig. 1) by PGMs/Mo substitution with both Fe^2+^ and Fe^3+^ sites.

Figure 1 shows (a) the experimental spectra with respect to the absorption energy before (black) and after (red) 24 h sorption test [the intensity (absorbance) of the two spectra was normalized at 6 eV], and (b–d) theoretical spectra of the PB model before and after substitution with Fe^2+^ or Fe^3+^ for PGMs/Mo ions. For the pristine PBNPs, the three absorption band around 1.6 eV (weak), 2.1 eV (intense), 3.1 eV (weak), and 5.0 eV (middle) originate from the charge-transfer (CT) transitions from Fe^2+^ to Fe^3+^, which correspond to peaks A, B, C, and D, respectively^28^. For Pd ion (right hand), the theoretical spectra of the Pd ion substituted with Fe^2+^ (c) well explain the experimental spectral change (red shift) when compared to the spectra of the Pd ion substituted with Fe^3+^ (d). This is consistent with the previous results obtained using CASTEP^24^. For Rh ion (right-middle hand), comparison in theoretical spectra between before (b) and after (c, d) Rh substitution indicates that spectra (d) obtained for Rh substituted with Fe^3+^ reasonably explain the experimental spectral change after Rh sorption test. Thus, it is suitable to be concluded that Rh ion is mainly substituted with Fe^3+^ ion. For Ru ion (left-middle hand), comparison in theoretical spectra between before (b) and after (c, d) Ru substitution indicates that spectra (d) obtained for Ru substituted with Fe^3+^ obviously explain the experimental spectral change after Ru sorption test, because no red-shift in spectra after Ru sorption test was observed experimentally. For Mo ion (left hand), since the experimental spectra changed toward both a lower and a higher energy region after Mo ion sorption, it is found from theoretical spectral changes before (b) and after (c, d) Mo substitution that Mo^6+^ ion is substituted with both Fe^2+^ and Fe^3+^ ions.

In summary, it is reasonably concluded from the results of Fig. 1 that Ru^4+^ and Rh^3+^ ions are substituted with Fe^3+^ ion, and Pd^2+^ ion is substituted with Fe^2+^ ion, whereas Mo^6+^ ion is substituted with both Fe^2+^ and Fe^3+^ ions.

### XRD

**Figure 2:**
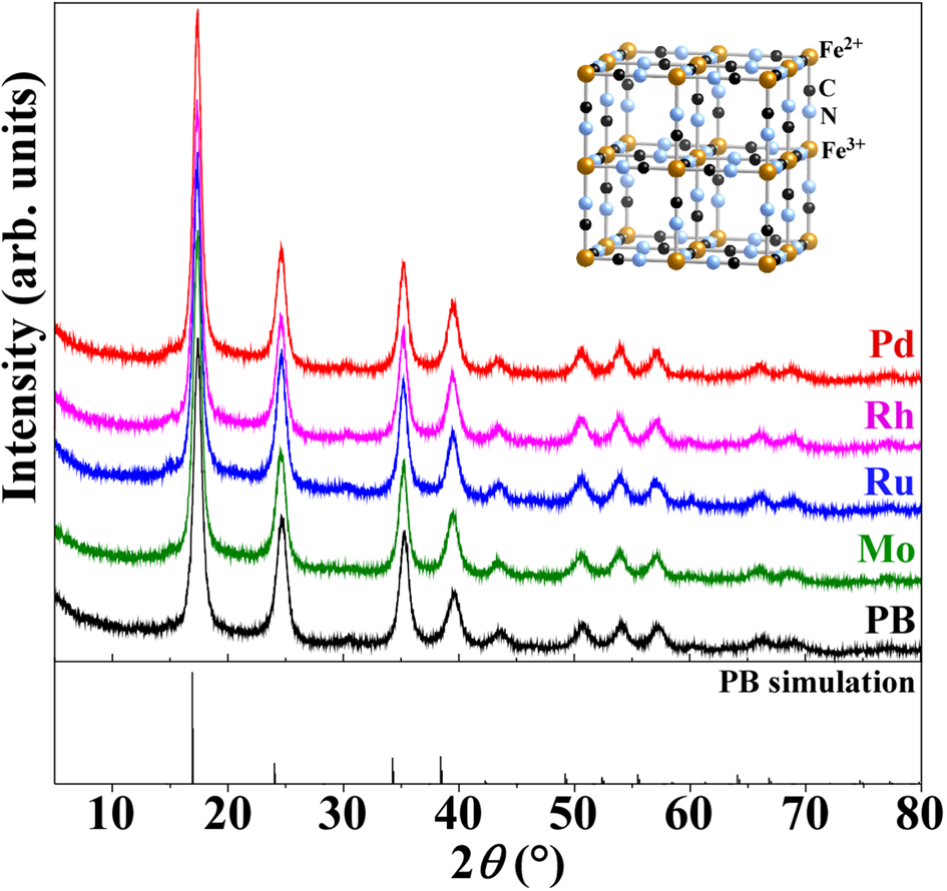
Powder XRD patterns of the pristine and PGMs/Mo-sorbed PBNPs, along with the simulation results (stick bar) of FCC structure (space group: $F\overline{4} 3m$) for PB. The black, green, blue, pink, and red lines represent the pristine, Mo-, Ru-, Rh-, and Pd-sorbed PBNPs, respectively. The crystal structure of PB is shown in the inset.

Figure 2 shows XRD patterns of the pristine (black) and PGM-sorbed (blue: Ru, pink: Rh, and red: Pd) PBNPs. Here, the inset shows the unit cell of PB crystal structure. The XRD pattern (black) shows that the pristine PBNPs have a face centered cubic (FCC) structure (space group: $F\overline{4} 3m$), which is consistent with the previous results obtained using high-resolution transmission electron microscope^24^. Table 2 summarizes the lattice constant and crystallite size of the PBNPs before and after 24 h PGMs/Mo sorption test, which were estimated by fitting the (200) diffraction peak with pseudo-Voigt function and by using the Scherrer equation^47^ with a constant of 1.5, respectively. Here, the constant value means an area-weighted effective diameter along the direction of the diffraction vector. The results of Fig. 2 and Table 2 indicate that PBNPs with a crystallite size of 16–17 nm were found to maintain the FCC structure before and after the PGMs/Mo sorption, and that the lattice constant was estimated to be 10.16 Å for the pristine PBNPs (10.13–10.18 Å range reported previously^48–50^), whereas 10.21 Å for the PGM/Mo-sorbed PBNPs.

**Table 2:** The crystallite size and lattice constant of the pristine and PGMs/Mo-sorbed PBNPs.

	Crystallite size (nm)	Lattice constant (Å)
Pristine PB	16.1	10.16
Ru-sorbed PB	16.3	10.21
Rh-sorbed PB	16.7	10.21
Pd-sorbed PB	16.7	10.21
Mo-sorbed PB	16.7	10.21

For Pd ion sorption, since the ionic radius of Pd^2+^ ion (0.86 Å)^46^ is larger than that of Fe^2+^ (0.61 Å)^46^ and the substitution efficiency of Fe^2+^ with Pd^2+^ was 87.0% (Table 1), the lattice constant should be expanded significantly. For Rh ion sorption, the ionic radius of Rh^3+^ ion (0.665 Å)^46^ is larger than that of Fe^3+^ (0.645 Å)^46^ to some extent, and also the substitution efficiency of Fe^3+^ with Rh^3+^ was 47.8%, thus the lattice constant was expanded effectively. On the contrary, for the Ru sorption, despite the ionic radius of Ru^4+^ ion (0.62 Å)^46^ is smaller than that of Fe^3+^ (0.645 Å) with 39.0% of the substitution efficiency of Fe^3+^ with Ru^4+^, the lattice was expanded in a similar manner to Pd ion sorption. This is presumably because the Coulomb repulsion between Ru^4+^ and Fe^2+^ is stronger than that between Fe^3+^ and Fe^2+^, resulting in the expansion of the lattice constant. In a similar manner for the Mo sorption, although the ionic radii of 0.59 Å for Mo^6+^ is smaller than that of Fe^3+^ (0.645 Å) with 17.1% of the substitution efficiency of Fe^2+^/Fe^3+^ with Ru^4+^, a larger Coulomb repulsion between Mo^6+^ and Fe^2+^/Fe^3+^ stronger than that between Fe^3+^ and Fe^2+^ caused the expansion of the lattice constant, which is comparable to that for the PGMs. The reason why the lattice constant was expanded to be coincidently the same value after sorption of all PGMs/Mo ions is still unclear. It is necessary to be considered that the residual PGMs/Mo and Fe ions located in the nanospace of PB unit cell upon sorption play a role in expanding the lattice constant.

### Elementary processes of PGMs sorption into PBNPs

To discuss the elementary processes of PGMs/Mo when incorporated into PBNPs, we next examined (1) the adsorption energy of PGMs/Mo ions on the PB surface, (2) the diffusion energy of PGMs/Mo ions into the nanospace of PBNPs, and (3) the substitution energy of PGMs/Mo ions with Fe ion, using first-principles calculations based on DFT.

**Figure 3:**
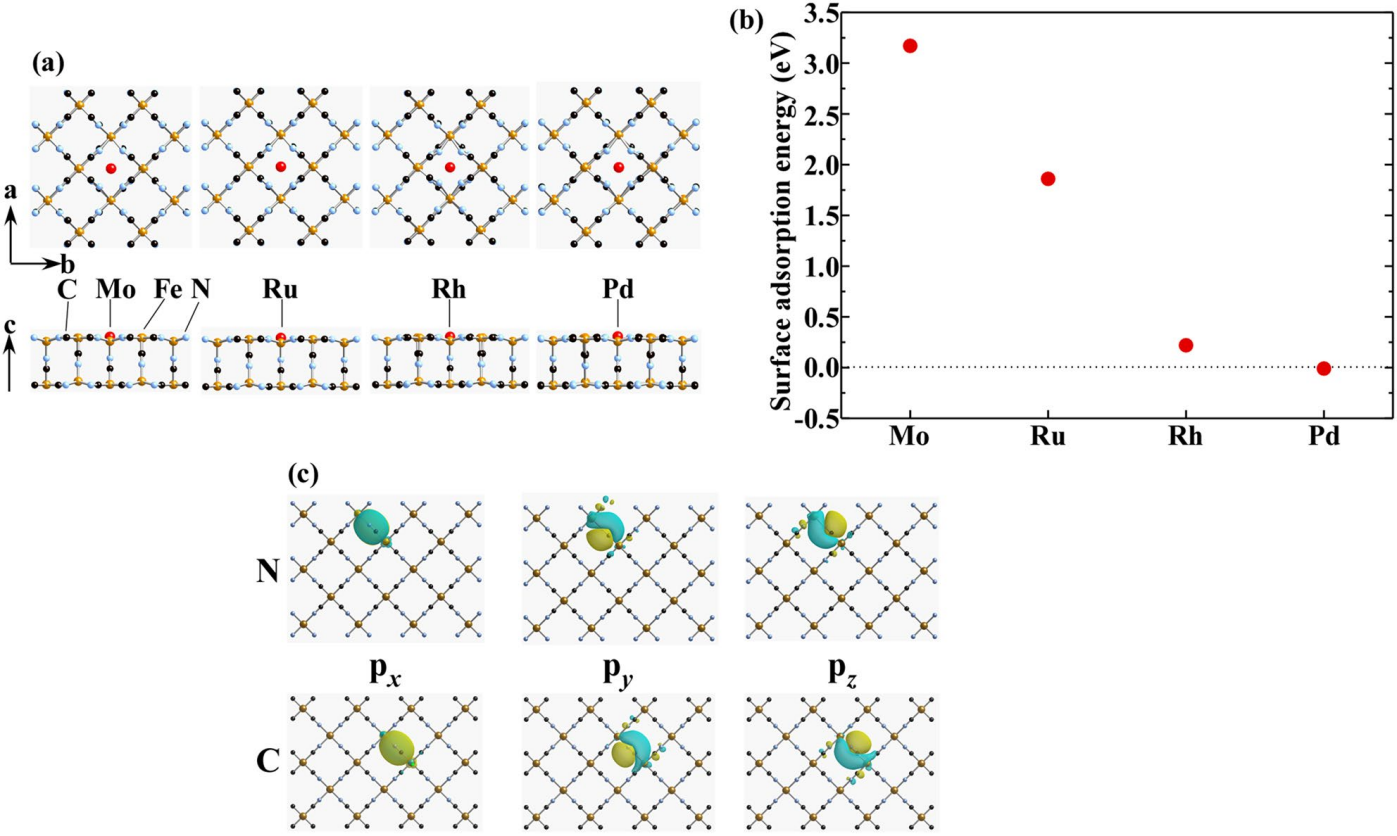
(**a**) Surface adsorption energy of PGMs/Mo bare ions on the (100) surface of PB, (**b**) the optimized surface structure of PGMs/Mo ions on the PB (100) surface, and (**c**) the Wannier function of the C2p and N2p atomic orbitals on the PB (100) surface (isosurface = 0.02).

We first discuss (1) the adsorption energy. Figure 3 shows (a) the optimized structure of PGMs/Mo ions on the (100) surface of PB, (b) the adsorption energy (*E*_ad_) of PGMs/Mo on the surface, and (c) the maximally-localized Wannier functions (MLWFs) of the C2p and N2p AOs for the CN group constructing the (100) surface. As shown in the side view of Fig. 3a, the (100) surface model was optimized to be the zig-zag structure which is stabler by 1.4 eV than the bulk flat surface structure. As shown in shown in the top view of Fig. 3a, it is found that all PGMs/Mo ions (red circle) are stably adsorbed on the center of the square consisting of the four Fe ions in the (100) plane. This is because PGMs/Mo ions surrounded by the four CN^–^ anion groups became stable energetically due to the attractive Coulomb interactions. The optimized structural coordination was summarized in Tables S1–S5 (supporting materials). Figure 3b shows the plot of the *E*_ad_ on the (100) surface with respect to the PGM elements. Here, a lager positive value of *E*_ad_ implies a more difficulty to be adsorbed on the surface. It is found that the *E*_ad_ seems to increase non-linearly with the valence number of PGMs ions: *E*_ad_ = 0 eV for Pd^2+^ ion, ca. 0.2 eV for Rh^3+^ ion, ca. 1.8 eV for Ru^4+^ ion, and ca. 3.2 eV for Mo^6+^. This is partly because the repulsive Coulomb interactions between the adsorbed PGMs/Mo ions and their surrounding Fe^2+^/Fe^3+^ ions become greater than the attractive ones between the adsorbed ions and their surrounding CN^–^ groups as the valence number of the adsorbed PGM/Mo ions increases, and partly because the latter attractive interactions become greater to cause a large distortion of the lattice with increasing the valence number as well. In addition, as show in in Fig. 3c, since the 2p_y_ and 2p_z_ AOs of both C and N atoms are expanded within the (100) in-plane, the PGMs/Mo cations are easy to be trapped in the (100) in-plane. It is noted that the present study estimated the *E*_ad_ in solid phase and in vacuum. For the practical HLLW system with metal ions in nitric acid solution, the metal ions are more easily transported to the PB surface via the solvent, and the electrostatic potential of the (100) surface should also be somewhat altered.

**Figure 4:**
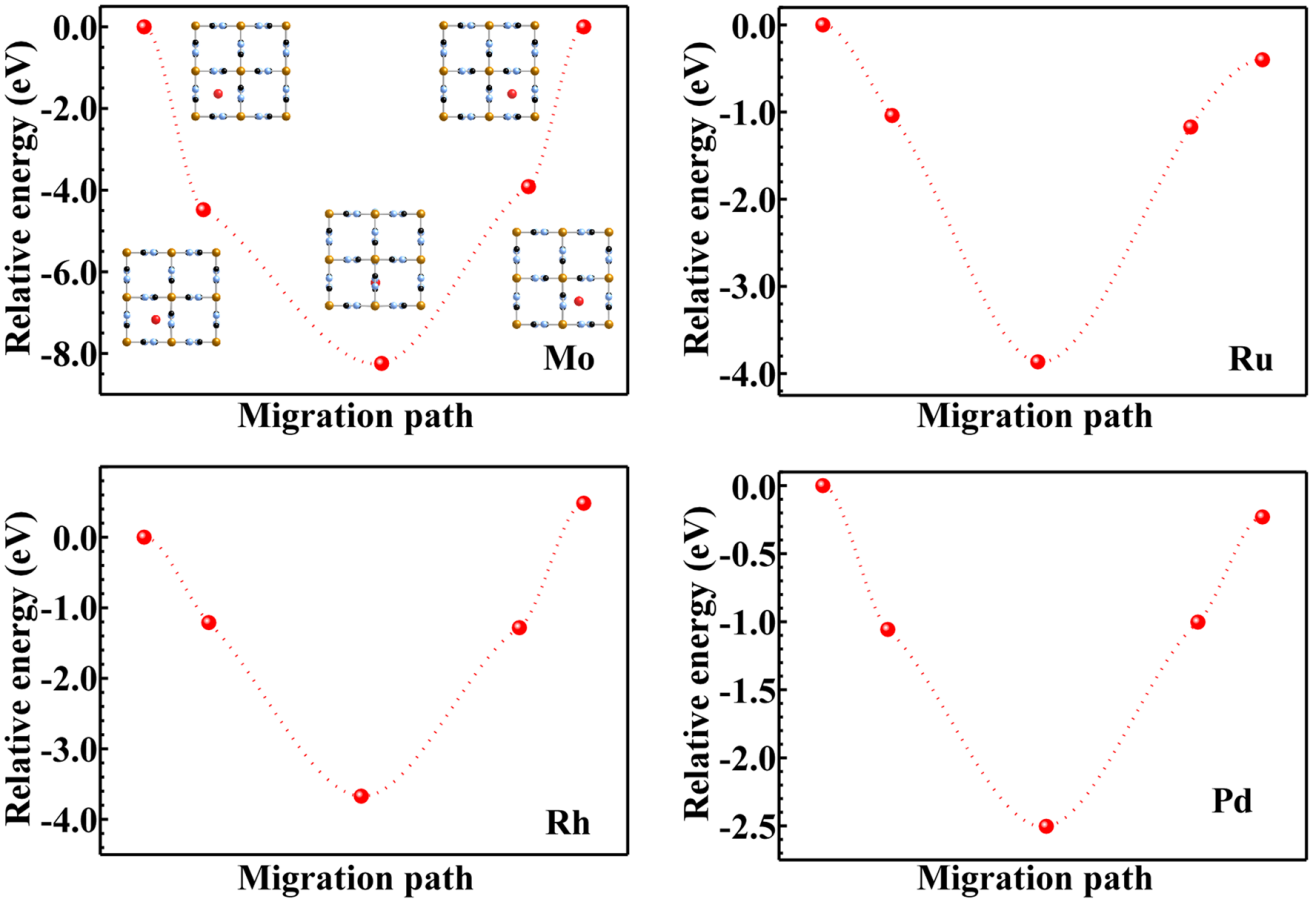
Relative energy of PGMs/Mo ions into PB jungle-gym structure at each migration step for the diffusion model. Inset shows the snap shot of each migration step in the nanospace of PB unit cell.

We next discuss (2) the diffusion of PGMs ions in the nanospace of PB unit cell, whose energy was calculated using the NEB method. Figure 4 shows the plot of the relative energy with respect to the five migration steps for PGMs/Mo. Inset schematically illustrates a snap shot at each migration step. The accurate structural parameters for each migration step were summarized in Tables S6.1–S9.5 (supporting materials). It is interesting to note that all PGMs/Mo ions become stable at the center of the square formed by the four Fe ions in the (100) plane, whereas the 4*c* site is a saddle point for those metal ions. The reason behind the stabilization of the PGMs/Mo ions in the (100) in-plane is the same as for their surface adsorption. In addition, it can be said that the space of the 4*c* site (5 Å) is too large for PGMs ions (Ionic diameter: 1.24 Å for Ru^4+^, 1.33 Å for Rh^3+^, 1.72 Å for Pd^2+^, 0.59 Å for Mo^6+^)^45^. Thus, the PGMs/Mo ions tend to be trapped at the (100) in-plane, where the substitution reaction between PGMs/Mo and Fe ions will take place.

**Figure 5:**
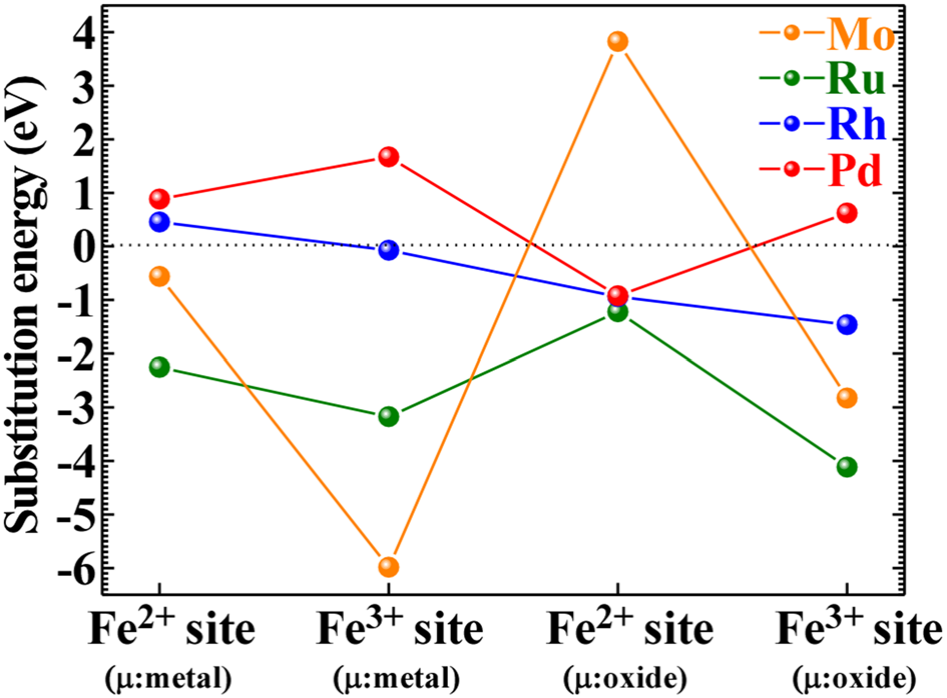
The substitution energy of PGMs/Mo ions with Fe^2+^/Fe^3+^ ions in PB in the reduction and oxidation limits, respectively. The horizontal axis represents the substitution site and the type of the chemical potential *μ* in parentheses.

We finally discuss the substitution energy (*E*_S_) of PGMs/Mo ions with the Fe^2+^/Fe^3+^ ions of PB, which was estimated using the unit cell model containing 60 atoms (inset of Fig. 2). Although it is supposed that the model corresponds to 12.5% Fe ions (one of eight) substituted with PGMs ions when applied to the solid state, we just focused on the substitution occurred locally using the unit cell. Figure 5 shows the *E*_S_ of PGMs/Mo ions with the Fe ions of PB unit cell. When the *E*_S_ is a negative value, the substitution takes place preferably. Since the *E*_S_ was defined by Eq. (2) described above, the chemical potentials were required. In the present study, we used the chemical potential of each metal oxide at the oxidation limit, and of neutral metal atom at the reduction limit. At the oxidation limit, Pd^2+^ and Ru^4+^ ions are expected to be substituted energetical preferentially with the Fe^2+^ and Fe^3+^ sites, respectively. On the other hand, Rh^3+^ ion is expected to be substituted with both sites because of the small difference (ca. 0.4 eV) in *E*_s_ between them. Although the results of Fig. 1 indicate that Rh^3+^ ion is substituted mainly with Fe^3+^ ion, Rh^3+^ ion would be substituted minorly with Fe^2+^ ion to some extent at temperatures around 353 K when applied to a practical HLLW containing the exothermic nuclides such as ^137^Cs and ^90^Sr. On the other hand, Mo^6+^ ion is preferable to be substituted with the Fe^3+^ site energetically, because the Coulomb repulsion between Mo^6+^ and the nearest surrounding Fe^3+^ ions for Mo^6+^ substitution with Fe^2+^ becomes much greater than that between Mo^6+^ and the nearest surrounding Fe^2+^ for Mo^6+^ substitution with Fe^3+^. However, comparison between the experimental and theoretical results (Fig. 1) indicates that Mo^6+^ is substituted not only with Fe^3+^ ion but also with Fe^2+^ ion, which is contradictory to that the *E*_s_ of Fe^2+^ with Mo^6+^ is a large positive value of ca. 4.0 eV at the oxidation limit, as shown in Fig. 5. Since the present PB unit cell cluster model did not consider the Madelung potentials and the lattice relaxation, the *E*_s_ may be overestimated when Fe^2+^/Fe^3+^ is substituted with the higher valent Mo^6+^ ion.

We examined the three elementary processes when PGMs ions are incorporated into PB: the adsorption on the (100) surface of PB, the diffusion in the nanospace of PB unit cell, and the substitution of PGMs ions with Fe ions. As summarized in Table 3, all the three processes are most easily to progress for Pd^2+^ ion, and subsequently for Rh^3+^ ion, whereas the surface adsorption process becomes the rate-determining step for Ru^4+^ ion. This is consistent with the results of the sorption and substitution efficiencies for these metal ions, as shown in Table 1. On the other hand, despite the order of the elusion and substitution efficiencies for Mo ion can be well explained using the results of Table 3 as well as PGMs ions, the sorption efficiency of Mo ion was higher than that for PGMs ions. Since Mo ion tends to be formed as polynuclear Mo oxide complexes in a nitric acid solution^29^, a part of them was presumably precipitated in the solution, which was not involved in the 24-h sorption test.

**Table 3:** The adsorption energy, diffusion barrier, and substitution energy of PGMs/Mo ions when incorporated into PB unit cells.

	Adsorption energy (eV)	Diffusion barrier (eV)	Substitution energy (eV)
Ru^4+^	1.8	4.0	– 1.2 (Fe^2+^)/– 4.1 (Fe^3+^)
Rh^3+^	0.2	3.8	– 0.9 (Fe^2+^)/– 1.5 (Fe^3+^)
Pd^2+^	0	2.5	– 0.9 (Fe^2+^)/+ 0.6 (Fe^3+^)
Mo^6+^	3.2	8.0	+ 3.8 (Fe^2+^)/– 2.8 (Fe^3+^)

The findings in the present study provide us an important insight that these three processes mutually affect the sorption amount and the sorption efficiency, thus leading to develop a high-performance sorbent for recovery of rare metals not only from N-wastes but also from E-wastes.

### The uptake amount of PGMs per 1 g PBNPs

As shown in Table 1, the uptake mechanism of PGMs/Mo ions when incorporated into PBNPs can allow us to estimate the substitution efficiency of PGMs/Mo with Fe by considering the PB unit cell (inset of Fig. 2) with inclusion of four Fe^2+^ and four Fe^3+^ atoms. Assuming that the amount of Fe elution is equal to that of PGMs/Mo substitution (namely, the amount of PGMs/Mo and Fe diffusion inside PBNPs is neglected), the substitution efficiency was obtained to be 39.0% for Ru ion, 47.8% for Rh ion, 87.0% for Pd ion, and 17.1% (Fe^2+^ and Fe^3+^ for each) for Mo ion. This implies that 0.13 g of Ru, 0.16 g of Rh, 0.30 g of Pd, and 0.11 g of Mo can be recovered by using 1 g PB with a chemical form of KFe(III)[Fe(II)(CN)_6_]. Furthermore, PBNPs were confirmed to exhibit several resistances against heat (up to 300 °C), nitric acid (up to 8 M), and γ-ray radiation (up to 1000 kGy), thus indicating that PBNPs are practically used not only for disposal processes of N-wastes but also for recycle processes of E-wastes.

## Summary

We have examined the uptake mechanisms of platinum-group-metals (PGMs) and molybdenum (Mo) ions into PBNPs in a nitric acid solution for 24-h sorption test, using ICP-AES, powder XRD, and UV–Vis-NIR spectroscopy in combination with first-principles calculations, and revealed that the Ru^4+^ and Pd^2+^ ions are incorporated into PBNPs by substitution with Fe^3+^ and Fe^2+^ ions of the PB framework, respectively, whereas the Rh^3+^ ion is incorporated into PBNPs by substitution mainly with Fe^3+^ and minorly with Fe^2+^ ion, and Mo^6+^ ion is incorporated into PBNPs by substitution with both Fe^2+^ and Fe^3+^ ions, with maintaining the crystal structure before and after the sorption test. Assuming that the amount of Fe elusion is equal to that of PGMs/Mo substitution, the substitution efficiency is estimated to be 39.0% for Ru, 47.8% for Rh, 87% for Pd, and 17.1% for Mo^6+^. This implies that 0.13 g of Ru, 0.16 g of Rh, 0.30 g of Pd, and 0.107 g of Mo can be recovered by using 1 g PBNPs with a chemical form of KFe(III)[Fe(II)(CN)_6_].

Since PBNPs can be produced massively in liquid phase and exhibit nontoxic and stable up to 300 °C, they are well known to be used as pigments and paint color materials. Thus, the present findings demonstrate that PB (or its analogues) will be one of the hopeful candidates to develop the recycling of precious metals from N- and E-wastes when compared to conventional bio-based adsorbents/activated carbons.

## Supplementary Information

Supplementary Information.
