# Supplementary material for: The uptake characteristics of Prussian-blue nanoparticles for rare metal ions for recycling of precious metals from nuclear and electronic wastes
Source: Sci Rep. 2022 Mar 24;12:5135. doi: 10.1038/s41598-022-08838-1 (PMC8948275; doi:10.1038/s41598-022-08838-1)
Supplement: Supplementary file 1 — Supplementary Information. [file 41598_2022_8838_MOESM1_ESM.pdf]

Supplementary Materials

The uptake characteristics of Prussian-blue nanoparticles for rare metal ions for recycling of precious metals from nuclear and electronic wastes

Shinta Watanabe, Yusuke Inaba, Miki Harigai, Kenji Takeshita, and Jun Onoe

Table S1. The fractional coordinates of the optimized structure for PB (100) surface. The lattice constant and angle were set to a, b = 7.1224 Å, c = 15.4436 Å, and α, β, γ = 90º, respectively.

Atom	x	y	z
Fe	0.00000	0.50000	0.14640
Fe	0.50000	0.00000	0.85360
Fe	0.50000	0.00000	0.16844
Fe	0.00000	0.50000	0.83156
C	0.50000	0.00000	0.05457
C	0.00000	0.50000	0.94543
C	0.68574	0.18574	0.17316
C	0.31426	0.81426	0.17316
C	0.31426	0.18574	0.17316
C	0.68574	0.81426	0.17316
C	0.81426	0.68574	0.82684
C	0.18574	0.31426	0.82684
C	0.18574	0.68574	0.82684
C	0.81426	0.31426	0.82684
N	0.50000	0.00000	0.97703
N	0.00000	0.50000	0.02297
N	0.80378	0.30378	0.17402
N	0.19622	0.69622	0.17402
N	0.19622	0.30378	0.17402
N	0.80378	0.69622	0.17402
N	0.69622	0.80378	0.82598

N	0.30378	0.19622	0.82598
N	0.30378	0.80378	0.82598
N	0.69622	0.19622	0.82598

Table S2. The fractional coordinates of the optimized structure for Mo on PB (100) surface. The lattice constant and angle were set to *a*, *b* = 14.2448 Å, *c* = 15.4436 Å, and α, β, γ = 90º, respectively.

Atom	*x*	*y*	*z*
C	0.34055	0.09590	0.17071
C	0.65945	0.90410	0.17071
C	0.34055	0.90410	0.17071
C	0.65945	0.09590	0.17071
C	0.14467	0.40335	0.16750
C	0.85533	0.59665	0.16750
C	0.14467	0.59665	0.16750
C	0.85533	0.40335	0.16750
C	0.15372	0.09358	0.16859
C	0.84628	0.90642	0.16859
C	0.15372	0.90642	0.16859
C	0.84628	0.09358	0.16859
C	0.33333	0.41432	0.17380
C	0.66667	0.58568	0.17380
C	0.33333	0.58568	0.17380
C	0.66667	0.41432	0.17380
C	0.40398	0.34599	0.82996
C	0.59602	0.65401	0.82996
C	0.40398	0.65401	0.82996
C	0.59602	0.34599	0.82996
C	0.09585	0.15409	0.82955
C	0.90415	0.84591	0.82955
C	0.09585	0.84591	0.82955
C	0.90415	0.15409	0.82955
C	0.09559	0.34569	0.82941

C	0.90441	0.65431	0.82941
C	0.09559	0.65431	0.82941
C	0.90441	0.34569	0.82941
C	0.40426	0.15449	0.83097
C	0.59574	0.84551	0.83097
C	0.40426	0.84551	0.83097
C	0.59574	0.15449	0.83097
C	0.00000	0.24812	0.94643
C	0.00000	0.75188	0.94643
C	0.24936	0.00000	0.05173
C	0.75064	0.00000	0.05173
C	0.50000	0.25284	0.94688
C	0.50000	0.74716	0.94688
C	0.24092	0.50000	0.05181
C	0.75908	0.50000	0.05181
N	0.39696	0.15775	0.16957
N	0.60304	0.84225	0.16957
N	0.39696	0.84225	0.16957
N	0.60304	0.15775	0.16957
N	0.09156	0.33926	0.16457
N	0.90844	0.66074	0.16457
N	0.09156	0.66074	0.16457
N	0.90844	0.33926	0.16457
N	0.09401	0.15172	0.16490
N	0.90599	0.84828	0.16490
N	0.09401	0.84828	0.16490
N	0.90599	0.15172	0.16490
N	0.40705	0.37176	0.17416
N	0.59295	0.62824	0.17416
N	0.40705	0.62824	0.17416
N	0.59295	0.37176	0.17416
N	0.34476	0.40490	0.83349
N	0.65524	0.59510	0.83349
N	0.34476	0.59510	0.83349
N	0.65524	0.40490	0.83349
N	0.15493	0.09509	0.83328

N	0.84507	0.90491	0.83328
N	0.15493	0.90491	0.83328
N	0.84507	0.09509	0.83328
N	0.15453	0.40491	0.83262
N	0.84547	0.59509	0.83262
N	0.15453	0.59509	0.83262
N	0.84547	0.40491	0.83262
N	0.34530	0.09535	0.83403
N	0.65470	0.90465	0.83403
N	0.34530	0.90465	0.83403
N	0.65470	0.09535	0.83403
N	0.00000	0.24580	0.02366
N	0.00000	0.75420	0.02366
N	0.24981	0.00000	0.97438
N	0.75019	0.00000	0.97438
N	0.50000	0.25608	0.02438
N	0.50000	0.74392	0.02438
N	0.24671	0.50000	0.97479
N	0.75329	0.50000	0.97479
Fe	0.00000	0.24420	0.14919
Fe	0.00000	0.75580	0.14919
Fe	0.00000	0.24985	0.83132
Fe	0.00000	0.75015	0.83132
Fe	0.24998	0.00000	0.84859
Fe	0.75002	0.00000	0.84859
Fe	0.24818	0.00000	0.16699
Fe	0.75182	0.00000	0.16699
Fe	0.50000	0.24701	0.14730
Fe	0.50000	0.75299	0.14730
Fe	0.50000	0.25054	0.83280
Fe	0.50000	0.74946	0.83280
Fe	0.24926	0.50000	0.84757
Fe	0.75074	0.50000	0.84757
Fe	0.23555	0.50000	0.16787
Fe	0.76445	0.50000	0.16787
Mo	0.50000	0.50000	0.18010

Table S3. The fractional coordinates of the optimized structure for Ru on PB (100) surface. The lattice constant and angle were set to a, b = 14.2448 Å, c = 15.4436 Å, and α, β, γ= 90º, respectively.

Atom	*x*	*y*	*z*
C	0.34731	0.09832	0.17121
C	0.65269	0.90168	0.17121
C	0.34731	0.90168	0.17121
C	0.65269	0.09832	0.17121
C	0.14816	0.40428	0.17142
C	0.85184	0.59572	0.17142
C	0.14816	0.59572	0.17142
C	0.85184	0.40428	0.17142
C	0.16295	0.08996	0.17151
C	0.83705	0.91004	0.17151
C	0.16295	0.91004	0.17151
C	0.83705	0.08996	0.17151
C	0.33660	0.41774	0.17448
C	0.66340	0.58226	0.17448
C	0.33660	0.58226	0.17448
C	0.66340	0.41774	0.17448
C	0.40674	0.33997	0.82723
C	0.59326	0.66003	0.82723
C	0.40674	0.66003	0.82723
C	0.59326	0.33997	0.82723
C	0.09582	0.15021	0.82782
C	0.90418	0.84979	0.82782
C	0.09582	0.84979	0.82782
C	0.90418	0.15021	0.82782
C	0.09255	0.33860	0.82505
C	0.90745	0.66140	0.82505
C	0.09255	0.66140	0.82505
C	0.90745	0.33860	0.82505
C	0.40458	0.15149	0.83143
C	0.59542	0.84851	0.83143
C	0.40458	0.84851	0.83143

C	0.59542	0.15149	0.83143
C	0.00000	0.24255	0.94282
C	0.00000	0.75745	0.94282
C	0.25642	0.00000	0.05292
C	0.74358	0.00000	0.05292
C	0.50000	0.25245	0.94587
C	0.50000	0.74755	0.94587
C	0.24084	0.50000	0.05410
C	0.75916	0.50000	0.05410
N	0.39886	0.16434	0.17091
N	0.60114	0.83566	0.17091
N	0.39886	0.83566	0.17091
N	0.60114	0.16434	0.17091
N	0.09518	0.34016	0.17073
N	0.90482	0.65984	0.17073
N	0.09518	0.65984	0.17073
N	0.90482	0.34016	0.17073
N	0.10000	0.14456	0.17036
N	0.90000	0.85544	0.17036
N	0.10000	0.85544	0.17036
N	0.90000	0.14456	0.17036
N	0.41181	0.37703	0.17412
N	0.58819	0.62297	0.17412
N	0.41181	0.62297	0.17412
N	0.58819	0.37703	0.17412
N	0.34874	0.40000	0.82911
N	0.65126	0.60000	0.82911
N	0.34874	0.60000	0.82911
N	0.65126	0.40000	0.82911
N	0.15650	0.09309	0.83342
N	0.84350	0.90691	0.83342
N	0.15650	0.90691	0.83342
N	0.84350	0.09309	0.83342
N	0.14981	0.39936	0.82691
N	0.85019	0.60064	0.82691
N	0.14981	0.60064	0.82691

N	0.85019	0.39936	0.82691
N	0.34449	0.09365	0.83456
N	0.65551	0.90635	0.83456
N	0.34449	0.90635	0.83456
N	0.65551	0.09365	0.83456
N	0.00000	0.24151	0.02011
N	0.00000	0.75849	0.02011
N	0.25255	0.00000	0.97552
N	0.74745	0.00000	0.97552
N	0.50000	0.26062	0.02313
N	0.50000	0.73938	0.02313
N	0.24454	0.50000	0.97699
N	0.75546	0.50000	0.97699
Fe	0.00000	0.23912	0.14403
Fe	0.00000	0.76088	0.14403
Fe	0.00000	0.24276	0.82835
Fe	0.00000	0.75724	0.82835
Fe	0.25029	0.00000	0.85054
Fe	0.74971	0.00000	0.85054
Fe	0.25908	0.00000	0.16746
Fe	0.74092	0.00000	0.16746
Fe	0.50000	0.25352	0.14577
Fe	0.50000	0.74648	0.14577
Fe	0.50000	0.24502	0.83240
Fe	0.50000	0.75498	0.83240
Fe	0.24811	0.50000	0.85131
Fe	0.75189	0.50000	0.85131
Fe	0.23833	0.50000	0.16967
Fe	0.76167	0.50000	0.16967
Ru	0.50000	0.50000	0.17693

Table S4. The fractional coordinates of the optimized structure for Rh on PB (100) surface. The lattice constant and angle were set to a, b = 14.2448 Å, c = 15.4436 Å, and α, β, γ= 90º, respectively.

Atom	*x*	*y*	*z*
C	0.34163	0.09387	0.17281
C	0.65837	0.90613	0.17281
C	0.34163	0.90613	0.17281
C	0.65837	0.09387	0.17281
C	0.15384	0.40686	0.17226
C	0.84616	0.59314	0.17226
C	0.15384	0.59314	0.17226
C	0.84616	0.40686	0.17226
C	0.15636	0.09276	0.17254
C	0.84364	0.90724	0.17254
C	0.15636	0.90724	0.17254
C	0.84364	0.09276	0.17254
C	0.34031	0.40850	0.17432
C	0.65969	0.59150	0.17432
C	0.34031	0.59150	0.17432
C	0.65969	0.40850	0.17432
C	0.40679	0.34291	0.82688
C	0.59321	0.65709	0.82688
C	0.40679	0.65709	0.82688
C	0.59321	0.34291	0.82688
C	0.09340	0.15638	0.82720
C	0.90660	0.84362	0.82720
C	0.09340	0.84362	0.82720
C	0.90660	0.15638	0.82720
C	0.09314	0.34297	0.82687
C	0.90686	0.65703	0.82687
C	0.09314	0.65703	0.82687
C	0.90686	0.34297	0.82687
C	0.40655	0.15637	0.82723
C	0.59345	0.84363	0.82723
C	0.40655	0.84363	0.82723

C	0.59345	0.15637	0.82723
C	0.00000	0.24967	0.94513
C	0.00000	0.75033	0.94513
C	0.25014	0.00000	0.05463
C	0.74986	0.00000	0.05463
C	0.50000	0.24992	0.94496
C	0.50000	0.75008	0.94496
C	0.24805	0.50000	0.05486
C	0.75195	0.50000	0.05486
N	0.39940	0.15426	0.17172
N	0.60060	0.84574	0.17172
N	0.39940	0.84574	0.17172
N	0.60060	0.15426	0.17172
N	0.09662	0.34625	0.17178
N	0.90338	0.65375	0.17178
N	0.09662	0.65375	0.17178
N	0.90338	0.34625	0.17178
N	0.09731	0.15165	0.17146
N	0.90269	0.84835	0.17146
N	0.09731	0.84835	0.17146
N	0.90269	0.15165	0.17146
N	0.40564	0.35530	0.17459
N	0.59436	0.64470	0.17459
N	0.40564	0.64470	0.17459
N	0.59436	0.35530	0.17459
N	0.34794	0.40205	0.82747
N	0.65206	0.59795	0.82747
N	0.34794	0.59795	0.82747
N	0.65206	0.40205	0.82747
N	0.15247	0.09746	0.82819
N	0.84753	0.90254	0.82819
N	0.15247	0.90254	0.82819
N	0.84753	0.09746	0.82819
N	0.15205	0.40205	0.82753
N	0.84795	0.59795	0.82753
N	0.15205	0.59795	0.82753

N	0.84795	0.40205	0.82753
N	0.34746	0.09748	0.82797
N	0.65254	0.90252	0.82797
N	0.34746	0.90252	0.82797
N	0.65254	0.09748	0.82797
N	0.00000	0.24963	0.02256
N	0.00000	0.75037	0.02256
N	0.25022	0.00000	0.97722
N	0.74978	0.00000	0.97722
N	0.50000	0.25087	0.02252
N	0.50000	0.74913	0.02252
N	0.24995	0.50000	0.97754
N	0.75005	0.50000	0.97754
Fe	0.00000	0.24857	0.14642
Fe	0.00000	0.75143	0.14642
Fe	0.00000	0.24959	0.83080
Fe	0.00000	0.75041	0.83080
Fe	0.24990	0.00000	0.85324
Fe	0.75010	0.00000	0.85324
Fe	0.24989	0.00000	0.16910
Fe	0.75011	0.00000	0.16910
Fe	0.50000	0.24514	0.14550
Fe	0.50000	0.75486	0.14550
Fe	0.50000	0.24956	0.83111
Fe	0.50000	0.75044	0.83111
Fe	0.24976	0.50000	0.85298
Fe	0.75024	0.50000	0.85298
Fe	0.24646	0.50000	0.16963
Fe	0.75354	0.50000	0.16963
Rh	0.50000	0.50000	0.18044

Table S5. The fractional coordinates of the optimized structure for Pd on PB (100) surface. The lattice constant and angle were set to a, b = 14.2448 Å, c = 15.4436 Å, and α, β, γ = 90º, respectively.

Atom	*x*	*y*	*z*
C	0.34333	0.09511	0.17289
C	0.65667	0.90489	0.17289
C	0.34333	0.90489	0.17289
C	0.65667	0.09511	0.17289
C	0.15357	0.40618	0.17270
C	0.84643	0.59382	0.17270
C	0.15357	0.59382	0.17270
C	0.84643	0.40618	0.17270
C	0.15839	0.09184	0.17333
C	0.84161	0.90816	0.17333
C	0.15839	0.90816	0.17333
C	0.84161	0.09184	0.17333
C	0.34013	0.41091	0.17443
C	0.65987	0.58909	0.17443
C	0.34013	0.58909	0.17443
C	0.65987	0.41091	0.17443
C	0.40687	0.34307	0.82691
C	0.59313	0.65693	0.82691
C	0.40687	0.65693	0.82691
C	0.59313	0.34307	0.82691
C	0.09304	0.15693	0.82656
C	0.90696	0.84307	0.82656
C	0.09304	0.84307	0.82656
C	0.90696	0.15693	0.82656
C	0.09302	0.34305	0.82645
C	0.90698	0.65695	0.82645
C	0.09302	0.65695	0.82645
C	0.90698	0.34305	0.82645
C	0.40691	0.15694	0.82710
C	0.59309	0.84306	0.82710
C	0.40691	0.84306	0.82710

C	0.59309	0.15694	0.82710
C	0.00000	0.24941	0.94427
C	0.00000	0.75059	0.94427
C	0.25189	0.00000	0.05539
C	0.74811	0.00000	0.05539
C	0.50000	0.25088	0.94510
C	0.50000	0.74912	0.94510
C	0.24676	0.50000	0.05537
C	0.75324	0.50000	0.05537
N	0.39947	0.15709	0.17195
N	0.60053	0.84291	0.17195
N	0.39947	0.84291	0.17195
N	0.60053	0.15709	0.17195
N	0.09684	0.34515	0.17273
N	0.90316	0.65485	0.17273
N	0.09684	0.65485	0.17273
N	0.90316	0.34515	0.17273
N	0.09826	0.14957	0.17239
N	0.90174	0.85043	0.17239
N	0.09826	0.85043	0.17239
N	0.90174	0.14957	0.17239
N	0.40681	0.35939	0.17446
N	0.59319	0.64061	0.17446
N	0.40681	0.64061	0.17446
N	0.59319	0.35939	0.17446
N	0.34791	0.40206	0.82710
N	0.65209	0.59794	0.82710
N	0.34791	0.59794	0.82710
N	0.65209	0.40206	0.82710
N	0.15200	0.09794	0.82733
N	0.84800	0.90206	0.82733
N	0.15200	0.90206	0.82733
N	0.84800	0.09794	0.82733
N	0.15199	0.40205	0.82693
N	0.84801	0.59795	0.82693
N	0.15199	0.59795	0.82693

N	0.84801	0.40205	0.82693
N	0.34793	0.09800	0.82702
N	0.65207	0.90200	0.82702
N	0.34793	0.90200	0.82702
N	0.65207	0.09800	0.82702
N	0.00000	0.24869	0.02168
N	0.00000	0.75131	0.02168
N	0.25072	0.00000	0.97798
N	0.74928	0.00000	0.97798
N	0.50000	0.25221	0.02268
N	0.50000	0.74779	0.02268
N	0.24899	0.50000	0.97803
N	0.75101	0.50000	0.97803
Fe	0.00000	0.24676	0.14525
Fe	0.00000	0.75324	0.14525
Fe	0.00000	0.24999	0.83019
Fe	0.00000	0.75001	0.83019
Fe	0.24988	0.00000	0.85424
Fe	0.75012	0.00000	0.85424
Fe	0.25264	0.00000	0.16961
Fe	0.74736	0.00000	0.16961
Fe	0.50000	0.24897	0.14558
Fe	0.50000	0.75103	0.14558
Fe	0.50000	0.25008	0.83144
Fe	0.50000	0.74992	0.83144
Fe	0.24970	0.50000	0.85387
Fe	0.75030	0.50000	0.85387
Fe	0.24494	0.50000	0.16979
Fe	0.75506	0.50000	0.16979
Pd	0.50000	0.50000	0.17797

Table S6-1. The fractional coordinates of the migration path 1 of the optimized structure for Mo-sorbed PB. The lattice constant and angle were set to *a*, *b*, *c* = 10.4841 Å and *α*, *β*, *γ* = 90º, respectively.

Atom	*x*	*y*	*z*
Mo	0.25000	0.25000	0.25000
Fe	0.50000	0.50000	0.50000
Fe	0.50000	0.00000	0.00000
Fe	0.00000	0.50000	0.00000
Fe	0.00000	0.00000	0.50000
Fe	0.00000	0.00000	0.00000
Fe	0.00000	0.50000	0.50000
Fe	0.50000	0.00000	0.50000
Fe	0.50000	0.50000	0.00000
N	0.29576	0.00000	0.00000
N	0.50000	0.29576	0.50000
N	0.50000	0.00000	0.20424
N	0.50000	0.20424	0.00000
N	0.00000	0.50000	0.20424
N	0.29576	0.50000	0.50000
N	0.50000	0.50000	0.29576
N	0.00000	0.20424	0.50000
N	0.00000	0.29576	0.00000
N	0.00000	0.00000	0.29576
N	0.20424	0.00000	0.50000
N	0.20424	0.50000	0.00000
N	0.70424	0.50000	0.50000
N	0.79576	0.00000	0.50000
N	0.79576	0.50000	0.00000
N	0.50000	0.00000	0.79576
N	0.00000	0.70424	0.00000
N	0.50000	0.79576	0.00000
N	0.00000	0.79576	0.50000
N	0.00000	0.50000	0.79576
N	0.50000	0.50000	0.70424
N	0.50000	0.70424	0.50000

N	0.70424	0.00000	0.00000
N	0.00000	0.00000	0.70424
C	0.18316	0.00000	0.00000
C	0.50000	0.18316	0.50000
C	0.50000	0.00000	0.31684
C	0.50000	0.31684	0.00000
C	0.00000	0.50000	0.31684
C	0.18316	0.50000	0.50000
C	0.50000	0.50000	0.18316
C	0.00000	0.31684	0.50000
C	0.00000	0.18316	0.00000
C	0.00000	0.00000	0.18316
C	0.31684	0.00000	0.50000
C	0.31684	0.50000	0.00000
C	0.81684	0.50000	0.50000
C	0.68316	0.00000	0.50000
C	0.68316	0.50000	0.00000
C	0.50000	0.00000	0.68316
C	0.00000	0.81684	0.00000
C	0.50000	0.68316	0.00000
C	0.00000	0.68316	0.50000
C	0.00000	0.50000	0.68316
C	0.50000	0.50000	0.81684
C	0.50000	0.81684	0.50000
C	0.81684	0.00000	0.00000
C	0.00000	0.00000	0.81684

Table S6-2. The fractional coordinates of the migration path 2 of the optimized structure for Mo-sorbed PB. The lattice constant and angle were set to *a*, *b*, *c* = 10.4841 Å, and α, β, γ = 90º, respectively.

Atom	*x*	*y*	*z*
Mo	0.25000	0.25000	0.31963
Fe	0.50415	0.50415	0.50158
Fe	0.99585	0.99585	0.50158
Fe	0.50145	0.99855	0.99797
Fe	0.99855	0.50145	0.99797
Fe	0.99941	0.99941	0.99848
Fe	0.50059	0.50059	0.99848
Fe	0.99838	0.50162	0.50071
Fe	0.50162	0.99838	0.50071
N	0.29667	0.00119	0.00110
N	0.49881	0.20333	0.00110
N	0.00119	0.29667	0.00110
N	0.20333	0.49881	0.00110
N	0.70228	0.49909	0.49964
N	0.79772	0.00091	0.49964
N	0.00091	0.79772	0.49964
N	0.49909	0.70228	0.49964
N	0.49027	0.29441	0.49199
N	0.29441	0.49027	0.49199
N	0.00973	0.20559	0.49199
N	0.20559	0.00973	0.49199
N	0.49802	0.00198	0.20351
N	0.00198	0.49802	0.20351
N	0.79740	0.49987	0.00002
N	0.00013	0.70260	0.00002
N	0.49987	0.79740	0.00002
N	0.70260	0.00013	0.00002
N	0.50005	0.99995	0.79710
N	0.99995	0.50005	0.79710
N	0.49736	0.49736	0.29582
N	0.00264	0.00264	0.29582
N	0.49922	0.49922	0.70144

N	0.00078	0.00078	0.70144
C	0.18433	0.99981	0.99935
C	0.50019	0.31567	0.99935
C	0.99981	0.18433	0.99935
C	0.31567	0.50019	0.99935
C	0.81453	0.50069	0.50023
C	0.68547	0.99931	0.50023
C	0.99931	0.68547	0.50023
C	0.50069	0.81453	0.50023
C	0.49725	0.18209	0.49704
C	0.18209	0.49725	0.49704
C	0.00275	0.31791	0.49704
C	0.31791	0.00275	0.49704
C	0.50076	0.99924	0.31566
C	0.99924	0.50076	0.31566
C	0.68503	0.50032	0.99929
C	0.99968	0.81497	0.99929
C	0.50032	0.68503	0.99929
C	0.81497	0.99968	0.99929
C	0.50081	0.99919	0.68491
C	0.99919	0.50081	0.68491
C	0.49930	0.49930	0.18332
C	0.00070	0.00070	0.18332
C	0.50007	0.50007	0.81401
C	0.99993	0.99993	0.81401

Table S6-3. The fractional coordinates of the migration path 3 of the optimized structure for Mo-sorbed PB. The lattice constant and angle were set to *a*, *b*, *c* = 10.4841 Å, and α, β, γ = 90º, respectively.

Atom	*x*	*y*	*z*
Mo	0.25000	0.25000	0.52447
Fe	0.51531	0.51531	0.50088
Fe	0.98469	0.98469	0.50088
Fe	0.50075	0.99925	0.00057
Fe	0.99925	0.50075	0.00057
Fe	0.00006	0.00006	0.00066
Fe	0.49994	0.49994	0.00066
Fe	0.99222	0.50778	0.50013
Fe	0.50778	0.99222	0.50013
N	0.29768	0.00017	0.99993
N	0.49983	0.20232	0.99993
N	0.00017	0.29768	0.99993
N	0.20232	0.49983	0.99993
N	0.70025	0.49598	0.49987
N	0.79975	0.00402	0.49987
N	0.00402	0.79975	0.49987
N	0.49598	0.70025	0.49987
N	0.45276	0.27467	0.51005
N	0.27467	0.45276	0.51005
N	0.04724	0.22533	0.51005
N	0.22533	0.04724	0.51005
N	0.50178	0.99822	0.20238
N	0.99822	0.50178	0.20238
N	0.79748	0.49999	0.00006
N	0.00001	0.70252	0.00006
N	0.49999	0.79748	0.00006
N	0.70252	0.00001	0.00006
N	0.50174	0.99826	0.79761
N	0.99826	0.50174	0.79761
N	0.49669	0.49669	0.30146
N	0.00331	0.00331	0.30146

N	0.49803	0.49803	0.70034
N	0.00197	0.00197	0.70034
C	0.18553	0.99971	0.00031
C	0.50029	0.31447	0.00031
C	0.99971	0.18553	0.00031
C	0.31447	0.50029	0.00031
C	0.81249	0.50284	0.49936
C	0.68751	0.99716	0.49936
C	0.99716	0.68751	0.49936
C	0.50284	0.81249	0.49936
C	0.49067	0.16646	0.50406
C	0.16646	0.49067	0.50406
C	0.00933	0.33354	0.50406
C	0.33354	0.00933	0.50406
C	0.50597	0.99403	0.31443
C	0.99403	0.50597	0.31443
C	0.68535	0.50011	0.00044
C	0.99989	0.81465	0.00044
C	0.50011	0.68535	0.00044
C	0.81465	0.99989	0.00044
C	0.50716	0.99284	0.68570
C	0.99284	0.50716	0.68570
C	0.49884	0.49884	0.18884
C	0.00116	0.00116	0.18884
C	0.49937	0.49937	0.81292
C	0.00063	0.00063	0.81292

Table S6-4. The fractional coordinates of the migration path 4 of the optimized structure for Mo-sorbed PB. The lattice constant and angle were set to a, b, c = 10.4841 Å, and α, β, γ = 90º, respectively.

Atom	x	y	z
Mo	0.25000	0.25000	0.68527
Fe	0.50447	0.50447	0.49848
Fe	0.99553	0.99553	0.49848
Fe	0.50150	0.99850	0.00224
Fe	0.99850	0.50150	0.00224
Fe	0.99939	0.99939	0.00127
Fe	0.50061	0.50061	0.00127
Fe	0.99858	0.50142	0.49943
Fe	0.50142	0.99858	0.49943
N	0.29653	0.00149	0.99871
N	0.49851	0.20347	0.99871
N	0.00149	0.29653	0.99871
N	0.20347	0.49851	0.99871
N	0.70235	0.49874	0.50035
N	0.79765	0.00126	0.50035
N	0.00126	0.79765	0.50035
N	0.49874	0.70235	0.50035
N	0.49139	0.29461	0.50801
N	0.29461	0.49139	0.50801
N	0.00861	0.20539	0.50801
N	0.20539	0.00861	0.50801
N	0.49990	0.00010	0.20305
N	0.00010	0.49990	0.20305
N	0.79727	0.50030	0.99993
N	0.99970	0.70273	0.99993
N	0.50030	0.79727	0.99993
N	0.70273	0.99970	0.99993
N	0.49784	0.00216	0.79655
N	0.00216	0.49784	0.79655
N	0.49923	0.49923	0.29828
N	0.00077	0.00077	0.29828

N	0.49763	0.49763	0.70424
N	0.00237	0.00237	0.70424
C	0.18421	0.00067	0.00054
C	0.49933	0.31579	0.00054
C	0.00067	0.18421	0.00054
C	0.31579	0.49933	0.00054
C	0.81476	0.49959	0.49977
C	0.68524	0.00041	0.49977
C	0.00041	0.68524	0.49977
C	0.49959	0.81476	0.49977
C	0.49845	0.18218	0.50309
C	0.18218	0.49845	0.50309
C	0.00155	0.31782	0.50309
C	0.31782	0.00155	0.50309
C	0.50061	0.99939	0.31534
C	0.99939	0.50061	0.31534
C	0.68494	0.50110	0.00063
C	0.99890	0.81506	0.00063
C	0.50110	0.68494	0.00063
C	0.81506	0.99890	0.00063
C	0.50049	0.99951	0.68430
C	0.99951	0.50049	0.68430
C	0.50006	0.50006	0.18588
C	0.99994	0.99994	0.18588
C	0.49960	0.49960	0.81664
C	0.00040	0.00040	0.81664

Table S6-5. The fractional coordinates of the migration path 5 of the optimized structure for Mo-sorbed PB. The lattice constant and angle were set to *a*, *b*, *c* = 10.4841 Å, and α, β, γ = 90°, respectively.

Atom	*x*	*y*	*z*
Mo	0.25000	0.25000	0.75000
Fe	0.50000	0.50000	0.50000
Fe	0.50000	0.00000	0.00000
Fe	0.00000	0.50000	0.00000
Fe	0.00000	0.00000	0.50000
Fe	0.00000	0.00000	0.00000
Fe	0.00000	0.50000	0.50000
Fe	0.50000	0.00000	0.50000
Fe	0.50000	0.50000	0.00000
N	0.29576	0.00000	0.00000
N	0.50000	0.29576	0.50000
N	0.50000	0.20424	0.00000
N	0.29576	0.50000	0.50000
N	0.50000	0.00000	0.79576
N	0.00000	0.50000	0.79576
N	0.50000	0.50000	0.70424
N	0.00000	0.20424	0.50000
N	0.00000	0.29576	0.00000
N	0.20424	0.00000	0.50000
N	0.00000	0.00000	0.70424
N	0.20424	0.50000	0.00000
N	0.70424	0.50000	0.50000
N	0.79576	0.00000	0.50000
N	0.50000	0.00000	0.20424
N	0.00000	0.50000	0.20424
N	0.79576	0.50000	0.00000
N	0.00000	0.70424	0.00000
N	0.50000	0.79576	0.00000
N	0.50000	0.50000	0.29576
N	0.00000	0.79576	0.50000
N	0.00000	0.00000	0.29576

N	0.50000	0.70424	0.50000
N	0.70424	0.00000	0.00000
C	0.18316	0.00000	0.00000
C	0.50000	0.18316	0.50000
C	0.50000	0.31684	0.00000
C	0.18316	0.50000	0.50000
C	0.50000	0.00000	0.68316
C	0.00000	0.50000	0.68316
C	0.50000	0.50000	0.81684
C	0.00000	0.31684	0.50000
C	0.00000	0.18316	0.00000
C	0.31684	0.00000	0.50000
C	0.00000	0.00000	0.81684
C	0.31684	0.50000	0.00000
C	0.81684	0.50000	0.50000
C	0.68316	0.00000	0.50000
C	0.50000	0.00000	0.31684
C	0.00000	0.50000	0.31684
C	0.68316	0.50000	0.00000
C	0.00000	0.81684	0.00000
C	0.50000	0.68316	0.00000
C	0.50000	0.50000	0.18316
C	0.00000	0.68316	0.50000
C	0.00000	0.00000	0.18316
C	0.50000	0.81684	0.50000
C	0.81684	0.00000	0.00000

Table S7-1. The fractional coordinates of the migration path 1 of the optimized structure for Ru-sorbed PB. The lattice constant and angle were set to a, b, c = 10.4841 Å, and α, β, γ = 90°, respectively.

Atom	x	y	z
Ru	0.25000	0.25000	0.25000
Fe	0.50000	0.50000	0.50000
Fe	0.50000	0.00000	0.00000
Fe	0.00000	0.50000	0.00000
Fe	0.00000	0.00000	0.50000
Fe	0.00000	0.00000	0.00000
Fe	0.00000	0.50000	0.50000
Fe	0.50000	0.00000	0.50000
Fe	0.50000	0.50000	0.00000
N	0.29576	0.00000	0.00000
N	0.50000	0.29576	0.50000
N	0.50000	0.00000	0.20424
N	0.50000	0.20424	0.00000
N	0.00000	0.50000	0.20424
N	0.29576	0.50000	0.50000
N	0.50000	0.50000	0.29576
N	0.00000	0.20424	0.50000
N	0.00000	0.29576	0.00000
N	0.00000	0.00000	0.29576
N	0.20424	0.00000	0.50000
N	0.20424	0.50000	0.00000
N	0.70424	0.50000	0.50000
N	0.79576	0.00000	0.50000
N	0.79576	0.50000	0.00000
N	0.50000	0.00000	0.79576
N	0.00000	0.70424	0.00000
N	0.50000	0.79576	0.00000
N	0.00000	0.79576	0.50000
N	0.00000	0.50000	0.79576
N	0.50000	0.50000	0.70424
N	0.50000	0.70424	0.50000

N	0.70424	0.00000	0.00000
N	0.00000	0.00000	0.70424
C	0.18316	0.00000	0.00000
C	0.50000	0.18316	0.50000
C	0.50000	0.00000	0.31684
C	0.50000	0.31684	0.00000
C	0.00000	0.50000	0.31684
C	0.18316	0.50000	0.50000
C	0.50000	0.50000	0.18316
C	0.00000	0.31684	0.50000
C	0.00000	0.18316	0.00000
C	0.00000	0.00000	0.18316
C	0.31684	0.00000	0.50000
C	0.31684	0.50000	0.00000
C	0.81684	0.50000	0.50000
C	0.68316	0.00000	0.50000
C	0.68316	0.50000	0.00000
C	0.50000	0.00000	0.68316
C	0.00000	0.81684	0.00000
C	0.50000	0.68316	0.00000
C	0.00000	0.68316	0.50000
C	0.00000	0.50000	0.68316
C	0.50000	0.50000	0.81684
C	0.50000	0.81684	0.50000
C	0.81684	0.00000	0.00000
C	0.00000	0.00000	0.81684

Table S7-2. The fractional coordinates of the migration path 2 of the optimized structure for Ru-sorbed PB. The lattice constant and angle were set to a, b, c = 10.4841 Å, and α, β, γ = 90º, respectively.

Atom	x	y	z
Ru	0.25000	0.25000	0.33288
Fe	0.50422	0.50422	0.50051
Fe	0.99578	0.99578	0.50051
Fe	0.50102	0.99898	0.99773
Fe	0.99898	0.50102	0.99773
Fe	0.99961	0.99961	0.99842
Fe	0.50039	0.50039	0.99842
Fe	0.99885	0.50115	0.49993
Fe	0.50115	0.99885	0.49993
N	0.29649	0.00077	0.00051
N	0.49923	0.20351	0.00051
N	0.00077	0.29649	0.00051
N	0.20351	0.49923	0.00051
N	0.70261	0.49880	0.49958
N	0.79739	0.00120	0.49958
N	0.00120	0.79739	0.49958
N	0.49880	0.70261	0.49958
N	0.48993	0.29427	0.49142
N	0.29427	0.48993	0.49142
N	0.01007	0.20573	0.49142
N	0.20573	0.01007	0.49142
N	0.49828	0.00172	0.20323
N	0.00172	0.49828	0.20323
N	0.79702	0.50001	0.99981
N	0.99999	0.70298	0.99981
N	0.50001	0.79702	0.99981
N	0.70298	0.99999	0.99981
N	0.49998	0.00002	0.79643
N	0.00002	0.49998	0.79643
N	0.49833	0.49833	0.29526
N	0.00167	0.00167	0.29526

N	0.49897	0.49897	0.70156
N	0.00103	0.00103	0.70156
C	0.18419	0.99992	0.99918
C	0.50008	0.31581	0.99918
C	0.99992	0.18419	0.99918
C	0.31581	0.50008	0.99918
C	0.81507	0.50021	0.49992
C	0.68493	0.99979	0.49992
C	0.99979	0.68493	0.49992
C	0.50021	0.81507	0.49992
C	0.49713	0.18176	0.49654
C	0.18176	0.49713	0.49654
C	0.00287	0.31824	0.49654
C	0.31824	0.00287	0.49654
C	0.50080	0.99920	0.31552
C	0.99920	0.50080	0.31552
C	0.68472	0.50025	0.99914
C	0.99975	0.81528	0.99914
C	0.50025	0.68472	0.99914
C	0.81528	0.99975	0.99914
C	0.50046	0.99954	0.68410
C	0.99954	0.50046	0.68410
C	0.49963	0.49963	0.18284
C	0.00037	0.00037	0.18284
C	0.49979	0.49979	0.81401
C	0.00021	0.00021	0.81401

Table S7-3. The fractional coordinates of the migration path 3 of the optimized structure for Ru-sorbed PB. The lattice constant and angle were set to a, b, c = 10.4841 Å, and α, β, γ = 90°, respectively.

Atom	*x*	*y*	*z*
Ru	0.25000	0.25000	0.49388
Fe	0.51087	0.51087	0.49940
Fe	0.98913	0.98913	0.49940
Fe	0.50049	0.99951	0.99977
Fe	0.99951	0.50049	0.99977
Fe	0.99998	0.99998	0.99967
Fe	0.50002	0.50002	0.99967
Fe	0.99399	0.50601	0.49970
Fe	0.50601	0.99399	0.49970
N	0.29706	0.00022	0.99995
N	0.49978	0.20294	0.99995
N	0.00022	0.29706	0.99995
N	0.20294	0.49978	0.99995
N	0.70011	0.49411	0.49995
N	0.79989	0.00589	0.49995
N	0.00589	0.79989	0.49995
N	0.49411	0.70011	0.49995
N	0.45108	0.27816	0.49682
N	0.27816	0.45108	0.49682
N	0.04892	0.22184	0.49682
N	0.22184	0.04892	0.49682
N	0.50181	0.99819	0.20306
N	0.99819	0.50181	0.20306
N	0.79698	0.50001	0.99988
N	0.99999	0.70302	0.99988
N	0.50001	0.79698	0.99988
N	0.70302	0.99999	0.99988
N	0.50163	0.99837	0.79652
N	0.99837	0.50163	0.79652
N	0.49855	0.49855	0.29878
N	0.00145	0.00145	0.29878

N	0.49785	0.49785	0.70032
N	0.00215	0.00215	0.70032
C	0.18476	0.99997	0.99982
C	0.50003	0.31524	0.99982
C	0.99997	0.18476	0.99982
C	0.31524	0.50003	0.99982
C	0.81240	0.50065	0.50002
C	0.68760	0.99935	0.50002
C	0.99935	0.68760	0.50002
C	0.50065	0.81240	0.50002
C	0.48574	0.16856	0.49842
C	0.16856	0.48574	0.49842
C	0.01426	0.33144	0.49842
C	0.33144	0.01426	0.49842
C	0.50586	0.99414	0.31499
C	0.99414	0.50586	0.31499
C	0.68470	0.50012	0.99975
C	0.99988	0.81530	0.99975
C	0.50012	0.68470	0.99975
C	0.81530	0.99988	0.99975
C	0.50532	0.99468	0.68455
C	0.99468	0.50532	0.68455
C	0.49944	0.49944	0.18595
C	0.00056	0.00056	0.18595
C	0.49911	0.49911	0.81316
C	0.00089	0.00089	0.81316

Table S7-4. The fractional coordinates of the migration path 4 of the optimized structure for Ru-sorbed PB. The lattice constant and angle were set to a, b, c = 10.4841 Å, and α, β, γ = 90º, respectively.

Atom	*x*	*y*	*z*
Ru	0.25000	0.25000	0.66402
Fe	0.50432	0.50432	0.49976
Fe	0.99568	0.99568	0.49976
Fe	0.50106	0.99894	0.00219
Fe	0.99894	0.50106	0.00219
Fe	0.99960	0.99960	0.00147
Fe	0.50040	0.50040	0.00147
Fe	0.99859	0.50141	0.50039
Fe	0.50141	0.99859	0.50039
N	0.29652	0.00069	0.99946
N	0.49931	0.20348	0.99946
N	0.00069	0.29652	0.99946
N	0.20348	0.49931	0.99946
N	0.70257	0.49877	0.50023
N	0.79743	0.00123	0.50023
N	0.00123	0.79743	0.50023
N	0.49877	0.70257	0.50023
N	0.48975	0.29443	0.50903
N	0.29443	0.48975	0.50903
N	0.01025	0.20557	0.50903
N	0.20557	0.01025	0.50903
N	0.49996	0.00004	0.20372
N	0.00004	0.49996	0.20372
N	0.79699	0.50000	0.00031
N	0.00000	0.70301	0.00031
N	0.50000	0.79699	0.00031
N	0.70301	0.00000	0.00031
N	0.49897	0.00103	0.79704
N	0.00103	0.49897	0.79704
N	0.49899	0.49899	0.29840
N	0.00101	0.00101	0.29840

N	0.49901	0.49901	0.70468
N	0.00099	0.00099	0.70468
C	0.18421	0.99986	0.00069
C	0.50014	0.31579	0.00069
C	0.99986	0.18421	0.00069
C	0.31579	0.50014	0.00069
C	0.81506	0.50006	0.49996
C	0.68494	0.99994	0.49996
C	0.99994	0.68494	0.49996
C	0.50006	0.81506	0.49996
C	0.49686	0.18192	0.50406
C	0.18192	0.49686	0.50406
C	0.00314	0.31808	0.50406
C	0.31808	0.00314	0.50406
C	0.50043	0.99957	0.31609
C	0.99957	0.50043	0.31609
C	0.68467	0.50018	0.00088
C	0.99982	0.81533	0.00088
C	0.50018	0.68467	0.00088
C	0.81533	0.99982	0.00088
C	0.50133	0.99867	0.68471
C	0.99867	0.50133	0.68471
C	0.49978	0.49978	0.18594
C	0.00022	0.00022	0.18594
C	0.49996	0.49996	0.81710
C	0.00004	0.00004	0.81710

Table S7-5. The fractional coordinates of the migration path 5 of the optimized structure for Ru-sorbed PB. The lattice constant and angle were set to a, b, c = 10.4841 Å, and α, β, γ = 90º, respectively.

Atom	x	y	z
Ru	0.25000	0.25000	0.75000
Fe	0.50000	0.50000	0.50000
Fe	0.50000	0.00000	0.00000
Fe	0.00000	0.50000	0.00000
Fe	0.00000	0.00000	0.50000
Fe	0.00000	0.00000	0.00000
Fe	0.00000	0.50000	0.50000
Fe	0.50000	0.00000	0.50000
Fe	0.50000	0.50000	0.00000
N	0.29576	0.00000	0.00000
N	0.50000	0.29576	0.50000
N	0.50000	0.20424	0.00000
N	0.29576	0.50000	0.50000
N	0.50000	0.00000	0.79576
N	0.00000	0.50000	0.79576
N	0.50000	0.50000	0.70424
N	0.00000	0.20424	0.50000
N	0.00000	0.29576	0.00000
N	0.20424	0.00000	0.50000
N	0.00000	0.00000	0.70424
N	0.20424	0.50000	0.00000
N	0.70424	0.50000	0.50000
N	0.79576	0.00000	0.50000
N	0.50000	0.00000	0.20424
N	0.00000	0.50000	0.20424
N	0.79576	0.50000	0.00000
N	0.00000	0.70424	0.00000
N	0.50000	0.79576	0.00000
N	0.50000	0.50000	0.29576
N	0.00000	0.79576	0.50000
N	0.00000	0.00000	0.29576

N	0.50000	0.70424	0.50000
N	0.70424	0.00000	0.00000
C	0.18316	0.00000	0.00000
C	0.50000	0.18316	0.50000
C	0.50000	0.31684	0.00000
C	0.18316	0.50000	0.50000
C	0.50000	0.00000	0.68316
C	0.00000	0.50000	0.68316
C	0.50000	0.50000	0.81684
C	0.00000	0.31684	0.50000
C	0.00000	0.18316	0.00000
C	0.31684	0.00000	0.50000
C	0.00000	0.00000	0.81684
C	0.31684	0.50000	0.00000
C	0.81684	0.50000	0.50000
C	0.68316	0.00000	0.50000
C	0.50000	0.00000	0.31684
C	0.00000	0.50000	0.31684
C	0.68316	0.50000	0.00000
C	0.00000	0.81684	0.00000
C	0.50000	0.68316	0.00000
C	0.50000	0.50000	0.18316
C	0.00000	0.68316	0.50000
C	0.00000	0.00000	0.18316
C	0.50000	0.81684	0.50000
C	0.81684	0.00000	0.00000

Table S8-1. The fractional coordinates of the migration path 1 of the optimized structure for Rh-sorbed PB. The lattice constant and angle were set to a, b, c = 10.4841 Å, and α, β, γ = 90º, respectively.

Atom	x	y	z
Ru	0.25000	0.25000	0.25000
Fe	0.50000	0.50000	0.50000
Fe	0.50000	0.00000	0.00000
Fe	0.00000	0.50000	0.00000
Fe	0.00000	0.00000	0.50000
Fe	0.00000	0.00000	0.00000
Fe	0.00000	0.50000	0.50000
Fe	0.50000	0.00000	0.50000
Fe	0.50000	0.50000	0.00000
N	0.29576	0.00000	0.00000
N	0.50000	0.29576	0.50000
N	0.50000	0.00000	0.20424
N	0.50000	0.20424	0.00000
N	0.00000	0.50000	0.20424
N	0.29576	0.50000	0.50000
N	0.50000	0.50000	0.29576
N	0.00000	0.20424	0.50000
N	0.00000	0.29576	0.00000
N	0.00000	0.00000	0.29576
N	0.20424	0.00000	0.50000
N	0.20424	0.50000	0.00000
N	0.70424	0.50000	0.50000
N	0.79576	0.00000	0.50000
N	0.79576	0.50000	0.00000
N	0.50000	0.00000	0.79576
N	0.00000	0.70424	0.00000
N	0.50000	0.79576	0.00000
N	0.00000	0.79576	0.50000
N	0.00000	0.50000	0.79576
N	0.50000	0.50000	0.70424
N	0.50000	0.70424	0.50000

N	0.70424	0.00000	0.00000
N	0.00000	0.00000	0.70424
C	0.18316	0.00000	0.00000
C	0.50000	0.18316	0.50000
C	0.50000	0.00000	0.31684
C	0.50000	0.31684	0.00000
C	0.00000	0.50000	0.31684
C	0.18316	0.50000	0.50000
C	0.50000	0.50000	0.18316
C	0.00000	0.31684	0.50000
C	0.00000	0.18316	0.00000
C	0.00000	0.00000	0.18316
C	0.31684	0.00000	0.50000
C	0.31684	0.50000	0.00000
C	0.81684	0.50000	0.50000
C	0.68316	0.00000	0.50000
C	0.68316	0.50000	0.00000
C	0.50000	0.00000	0.68316
C	0.00000	0.81684	0.00000
C	0.50000	0.68316	0.00000
C	0.00000	0.68316	0.50000
C	0.00000	0.50000	0.68316
C	0.50000	0.50000	0.81684
C	0.50000	0.81684	0.50000
C	0.81684	0.00000	0.00000
C	0.00000	0.00000	0.81684

Table S8-2. The fractional coordinates of the migration path 2 of the optimized structure for Rh-sorbed PB. The lattice constant and angle were set to a, b, c = 10.4841 Å, and α, β, γ = 90º, respectively.

Atom	*x*	*y*	*z*
Ru	0.25000	0.25000	0.32726
Fe	0.50332	0.50332	0.50021
Fe	0.99668	0.99668	0.50021
Fe	0.50088	0.99912	0.99837
Fe	0.99912	0.50088	0.99837
Fe	0.99974	0.99974	0.99867
Fe	0.50026	0.50026	0.99867
Fe	0.99878	0.50122	0.49981
Fe	0.50122	0.99878	0.49981
N	0.29627	0.00070	0.00061
N	0.49930	0.20373	0.00061
N	0.00070	0.29627	0.00061
N	0.20373	0.49930	0.00061
N	0.70280	0.49869	0.49973
N	0.79720	0.00131	0.49973
N	0.00131	0.79720	0.49973
N	0.49869	0.70280	0.49973
N	0.49130	0.29418	0.49192
N	0.29418	0.49130	0.49192
N	0.00870	0.20582	0.49192
N	0.20582	0.00870	0.49192
N	0.49902	0.00098	0.20349
N	0.00098	0.49902	0.20349
N	0.79667	0.49998	0.99982
N	0.00002	0.70333	0.99982
N	0.49998	0.79667	0.99982
N	0.70333	0.00002	0.99982
N	0.49996	0.00004	0.79625
N	0.00004	0.49996	0.79625
N	0.49893	0.49893	0.29513
N	0.00107	0.00107	0.29513

N	0.49892	0.49892	0.70204
N	0.00108	0.00108	0.70204
C	0.18388	0.99998	0.99942
C	0.50002	0.31612	0.99942
C	0.99998	0.18388	0.99942
C	0.31612	0.50002	0.99942
C	0.81531	0.50001	0.50002
C	0.68469	0.99999	0.50002
C	0.99999	0.68469	0.50002
C	0.50001	0.81531	0.50002
C	0.49706	0.18155	0.49624
C	0.18155	0.49706	0.49624
C	0.00294	0.31845	0.49624
C	0.31845	0.00294	0.49624
C	0.50100	0.99900	0.31588
C	0.99900	0.50100	0.31588
C	0.68429	0.50018	0.99924
C	0.99982	0.81571	0.99924
C	0.50018	0.68429	0.99924
C	0.81571	0.99982	0.99924
C	0.50041	0.99959	0.68384
C	0.99959	0.50041	0.68384
C	0.49984	0.49984	0.18266
C	0.00016	0.00016	0.18266
C	0.49971	0.49971	0.81454
C	0.00029	0.00029	0.81454

Table S8-3. The fractional coordinates of the migration path 3 of the optimized structure for Rh-sorbed PB. The lattice constant and angle were set to a, b, c = 10.4841 Å, and α, β, γ = 90°, respectively.

Atom	*x*	*y*	*z*
Ru	0.25000	0.25000	0.49671
Fe	0.50725	0.50725	0.49922
Fe	0.99275	0.99275	0.49922
Fe	0.50042	0.99958	0.99970
Fe	0.99958	0.50042	0.99970
Fe	0.00030	0.00030	0.99956
Fe	0.49970	0.49970	0.99956
Fe	0.99279	0.50721	0.49957
Fe	0.50721	0.99279	0.49957
N	0.29700	0.00016	0.99988
N	0.49984	0.20300	0.99988
N	0.00016	0.29700	0.99988
N	0.20300	0.49984	0.99988
N	0.69909	0.49327	0.49985
N	0.80091	0.00673	0.49985
N	0.00673	0.80091	0.49985
N	0.49327	0.69909	0.49985
N	0.45278	0.27838	0.49767
N	0.27838	0.45278	0.49767
N	0.04722	0.22162	0.49767
N	0.22162	0.04722	0.49767
N	0.50258	0.99742	0.20321
N	0.99742	0.50258	0.20321
N	0.79649	0.49993	0.99970
N	0.00007	0.70351	0.99970
N	0.49993	0.79649	0.99970
N	0.70351	0.00007	0.99970
N	0.50216	0.99784	0.79608
N	0.99784	0.50216	0.79608
N	0.49797	0.49797	0.29795
N	0.00203	0.00203	0.29795

N	0.49709	0.49709	0.70089
N	0.00291	0.00291	0.70089
C	0.18461	0.00015	0.99975
C	0.49985	0.31539	0.99975
C	0.00015	0.18461	0.99975
C	0.31539	0.49985	0.99975
C	0.81144	0.50090	0.49987
C	0.68856	0.99910	0.49987
C	0.99910	0.68856	0.49987
C	0.50090	0.81144	0.49987
C	0.48636	0.16882	0.49866
C	0.16882	0.48636	0.49866
C	0.01364	0.33118	0.49866
C	0.33118	0.01364	0.49866
C	0.50656	0.99344	0.31520
C	0.99344	0.50656	0.31520
C	0.68412	0.49991	0.99957
C	0.00009	0.81588	0.99957
C	0.49991	0.68412	0.99957
C	0.81588	0.00009	0.99957
C	0.50597	0.99403	0.68406
C	0.99403	0.50597	0.68406
C	0.49908	0.49908	0.18518
C	0.00092	0.00092	0.18518
C	0.49859	0.49859	0.81367
C	0.00141	0.00141	0.81367

Table S8-4. The fractional coordinates of the migration path 4 of the optimized structure for Rh-sorbed PB. The lattice constant and angle were set to a, b, c = 10.4841 Å, and α, β, γ = 90º, respectively.

Atom	x	y	z
Ru	0.25000	0.25000	0.67130
Fe	0.50326	0.50326	0.49950
Fe	0.99674	0.99674	0.49950
Fe	0.50088	0.99912	0.00194
Fe	0.99912	0.50088	0.00194
Fe	0.99970	0.99970	0.00122
Fe	0.50030	0.50030	0.00122
Fe	0.99920	0.50080	0.50031
Fe	0.50080	0.99920	0.50031
N	0.29629	0.00075	0.99957
N	0.49925	0.20371	0.99957
N	0.00075	0.29629	0.99957
N	0.20371	0.49925	0.99957
N	0.70308	0.49899	0.50036
N	0.79692	0.00101	0.50036
N	0.00101	0.79692	0.50036
N	0.49899	0.70308	0.50036
N	0.49307	0.29503	0.50619
N	0.29503	0.49307	0.50619
N	0.00693	0.20497	0.50619
N	0.20497	0.00693	0.50619
N	0.50002	0.99998	0.20389
N	0.99998	0.50002	0.20389
N	0.79674	0.49999	0.00018
N	0.00001	0.70326	0.00018
N	0.49999	0.79674	0.00018
N	0.70326	0.00001	0.00018
N	0.49858	0.00142	0.79672
N	0.00142	0.49858	0.79672
N	0.49930	0.49930	0.29779
N	0.00070	0.00070	0.29779

N	0.49840	0.49840	0.70472
N	0.00160	0.00160	0.70472
C	0.18389	0.99997	0.00068
C	0.50003	0.31611	0.00068
C	0.99997	0.18389	0.00068
C	0.31611	0.50003	0.00068
C	0.81560	0.50002	0.50011
C	0.68440	0.99998	0.50011
C	0.99998	0.68440	0.50011
C	0.50002	0.81560	0.50011
C	0.49792	0.18245	0.50261
C	0.18245	0.49792	0.50261
C	0.00208	0.31755	0.50261
C	0.31755	0.00208	0.50261
C	0.50035	0.99965	0.31632
C	0.99965	0.50035	0.31632
C	0.68435	0.50021	0.00069
C	0.99979	0.81565	0.00069
C	0.50021	0.68435	0.00069
C	0.81565	0.99979	0.00069
C	0.50054	0.99946	0.68430
C	0.99946	0.50054	0.68430
C	0.49992	0.49992	0.18530
C	0.00008	0.00008	0.18530
C	0.49955	0.49955	0.81721
C	0.00045	0.00045	0.81721

Table S8-5. The fractional coordinates of the migration path 5 of the optimized structure for Rh-sorbed PB. The lattice constant and angle were set to a, b, c = 10.4841 Å, and α, β, γ = 90°, respectively.

Atom	x	y	z
Ru	0.25000	0.25000	0.75000
Fe	0.50000	0.50000	0.50000
Fe	0.50000	0.00000	0.00000
Fe	0.00000	0.50000	0.00000
Fe	0.00000	0.00000	0.50000
Fe	0.00000	0.00000	0.00000
Fe	0.00000	0.50000	0.50000
Fe	0.50000	0.00000	0.50000
Fe	0.50000	0.50000	0.00000
N	0.29576	0.00000	0.00000
N	0.50000	0.29576	0.50000
N	0.50000	0.20424	0.00000
N	0.29576	0.50000	0.50000
N	0.50000	0.00000	0.79576
N	0.00000	0.50000	0.79576
N	0.50000	0.50000	0.70424
N	0.00000	0.20424	0.50000
N	0.00000	0.29576	0.00000
N	0.20424	0.00000	0.50000
N	0.00000	0.00000	0.70424
N	0.20424	0.50000	0.00000
N	0.70424	0.50000	0.50000
N	0.79576	0.00000	0.50000
N	0.50000	0.00000	0.20424
N	0.00000	0.50000	0.20424
N	0.79576	0.50000	0.00000
N	0.00000	0.70424	0.00000
N	0.50000	0.79576	0.00000
N	0.50000	0.50000	0.29576
N	0.00000	0.79576	0.50000
N	0.00000	0.00000	0.29576

N	0.50000	0.70424	0.50000
N	0.70424	0.00000	0.00000
C	0.18316	0.00000	0.00000
C	0.50000	0.18316	0.50000
C	0.50000	0.31684	0.00000
C	0.18316	0.50000	0.50000
C	0.50000	0.00000	0.68316
C	0.00000	0.50000	0.68316
C	0.50000	0.50000	0.81684
C	0.00000	0.31684	0.50000
C	0.00000	0.18316	0.00000
C	0.31684	0.00000	0.50000
C	0.00000	0.00000	0.81684
C	0.31684	0.50000	0.00000
C	0.81684	0.50000	0.50000
C	0.68316	0.00000	0.50000
C	0.50000	0.00000	0.31684
C	0.00000	0.50000	0.31684
C	0.68316	0.50000	0.00000
C	0.00000	0.81684	0.00000
C	0.50000	0.68316	0.00000
C	0.50000	0.50000	0.18316
C	0.00000	0.68316	0.50000
C	0.00000	0.00000	0.18316
C	0.50000	0.81684	0.50000
C	0.81684	0.00000	0.00000

Table S9-1. The fractional coordinates of the migration path 1 of the optimized structure for Pd-sorbed PB. The lattice constant and angle were set to a, b, c = 10.4841 Å, and α, β, γ = 90°, respectively.

Atom	x	y	z
Pd	0.25000	0.25000	0.25000
Fe	0.50000	0.50000	0.50000
Fe	0.50000	0.00000	0.00000
Fe	0.00000	0.50000	0.00000
Fe	0.00000	0.00000	0.50000
Fe	0.00000	0.00000	0.00000
Fe	0.00000	0.50000	0.50000
Fe	0.50000	0.00000	0.50000
Fe	0.50000	0.50000	0.00000
N	0.29576	0.00000	0.00000
N	0.50000	0.29576	0.50000
N	0.50000	0.00000	0.20424
N	0.50000	0.20424	0.00000
N	0.00000	0.50000	0.20424
N	0.29576	0.50000	0.50000
N	0.50000	0.50000	0.29576
N	0.00000	0.20424	0.50000
N	0.00000	0.29576	0.00000
N	0.00000	0.00000	0.29576
N	0.20424	0.00000	0.50000
N	0.20424	0.50000	0.00000
N	0.70424	0.50000	0.50000
N	0.79576	0.00000	0.50000
N	0.79576	0.50000	0.00000
N	0.50000	0.00000	0.79576
N	0.00000	0.70424	0.00000
N	0.50000	0.79576	0.00000
N	0.00000	0.79576	0.50000
N	0.00000	0.50000	0.79576
N	0.50000	0.50000	0.70424
N	0.50000	0.70424	0.50000

N	0.70424	0.00000	0.00000
N	0.00000	0.00000	0.70424
C	0.18316	0.00000	0.00000
C	0.50000	0.18316	0.50000
C	0.50000	0.00000	0.31684
C	0.50000	0.31684	0.00000
C	0.00000	0.50000	0.31684
C	0.18316	0.50000	0.50000
C	0.50000	0.50000	0.18316
C	0.00000	0.31684	0.50000
C	0.00000	0.18316	0.00000
C	0.00000	0.00000	0.18316
C	0.31684	0.00000	0.50000
C	0.31684	0.50000	0.00000
C	0.81684	0.50000	0.50000
C	0.68316	0.00000	0.50000
C	0.68316	0.50000	0.00000
C	0.50000	0.00000	0.68316
C	0.00000	0.81684	0.00000
C	0.50000	0.68316	0.00000
C	0.00000	0.68316	0.50000
C	0.00000	0.50000	0.68316
C	0.50000	0.50000	0.81684
C	0.50000	0.81684	0.50000
C	0.81684	0.00000	0.00000
C	0.00000	0.00000	0.81684

Table S9-2. The fractional coordinates of the migration path 2 of the optimized structure for Pd-sorbed PB. The lattice constant and angle were set to a, b, c = 10.4841 Å, and α, β, γ = 90°, respectively.

Atom	x	y	z
Pd	0.25000	0.25000	0.32372
Fe	0.50206	0.50206	0.50022
Fe	0.99794	0.99794	0.50022
Fe	0.50052	0.99948	0.99898
Fe	0.99948	0.50052	0.99898
Fe	0.99981	0.99981	0.99926
Fe	0.50019	0.50019	0.99926
Fe	0.99935	0.50065	0.49992
Fe	0.50065	0.99935	0.49992
N	0.29605	0.00054	0.00049
N	0.49946	0.20395	0.00049
N	0.00054	0.29605	0.00049
N	0.20395	0.49946	0.00049
N	0.70344	0.49921	0.49984
N	0.79656	0.00079	0.49984
N	0.00079	0.79656	0.49984
N	0.49921	0.70344	0.49984
N	0.49469	0.29500	0.49532
N	0.29500	0.49469	0.49532
N	0.00531	0.20500	0.49532
N	0.20500	0.00531	0.49532
N	0.49913	0.00087	0.20380
N	0.00087	0.49913	0.20380
N	0.79635	0.49996	0.00004
N	0.00004	0.70365	0.00004
N	0.49996	0.79635	0.00004
N	0.70365	0.00004	0.00004
N	0.49994	0.00006	0.79606
N	0.00006	0.49994	0.79606
N	0.49916	0.49916	0.29542
N	0.00084	0.00084	0.29542

N	0.49947	0.49947	0.70299
N	0.00053	0.00053	0.70299
C	0.18359	0.00003	0.99973
C	0.49997	0.31641	0.99973
C	0.00003	0.18359	0.99973
C	0.31641	0.49997	0.99973
C	0.81596	0.50003	0.50005
C	0.68404	0.99997	0.50005
C	0.99997	0.68404	0.50005
C	0.50003	0.81596	0.50005
C	0.49798	0.18240	0.49771
C	0.18240	0.49798	0.49771
C	0.00202	0.31760	0.49771
C	0.31760	0.00202	0.49771
C	0.50047	0.99953	0.31625
C	0.99953	0.50047	0.31625
C	0.68390	0.50011	0.99966
C	0.99989	0.81610	0.99966
C	0.50011	0.68390	0.99966
C	0.81610	0.99989	0.99966
C	0.50024	0.99976	0.68361
C	0.99976	0.50024	0.68361
C	0.49976	0.49976	0.18293
C	0.00024	0.00024	0.18293
C	0.49991	0.49991	0.81549
C	0.00009	0.00009	0.81549

Table S9-3. The fractional coordinates of the migration path 3 of the optimized structure for Pd-sorbed PB. The lattice constant and angle were set to a, b, c = 10.4841 Å, and α, β, γ = 90º, respectively.

Atom	x	y	z
Pd	0.25000	0.25000	0.50424
Fe	0.50563	0.50563	0.50010
Fe	0.99437	0.99437	0.50010
Fe	0.50015	0.99985	0.00003
Fe	0.99985	0.50015	0.00003
Fe	0.00006	0.00006	0.00006
Fe	0.49994	0.49994	0.00006
Fe	0.99712	0.50288	0.50004
Fe	0.50288	0.99712	0.50004
N	0.29642	0.00011	0.99999
N	0.49989	0.20358	0.99999
N	0.00011	0.29642	0.99999
N	0.20358	0.49989	0.99999
N	0.70256	0.49699	0.49999
N	0.79744	0.00301	0.49999
N	0.00301	0.79744	0.49999
N	0.49699	0.70256	0.49999
N	0.47867	0.29080	0.50087
N	0.29080	0.47867	0.50087
N	0.02133	0.20920	0.50087
N	0.20920	0.02133	0.50087
N	0.50068	0.99932	0.20379
N	0.99932	0.50068	0.20379
N	0.79630	0.49993	0.00001
N	0.00007	0.70370	0.00001
N	0.49993	0.79630	0.00001
N	0.70370	0.00007	0.00001
N	0.50069	0.99931	0.79626
N	0.99931	0.50069	0.79626
N	0.50011	0.50011	0.29707
N	0.99989	0.99989	0.29707

N	0.50023	0.50023	0.70308
N	0.99977	0.99977	0.70308
C	0.18396	0.00006	0.00002
C	0.49995	0.31604	0.00002
C	0.00006	0.18396	0.00002
C	0.31604	0.49995	0.00002
C	0.81521	0.50010	0.49999
C	0.68479	0.99990	0.49999
C	0.99990	0.68479	0.49999
C	0.50010	0.81521	0.49999
C	0.49316	0.17825	0.50040
C	0.17825	0.49316	0.50040
C	0.00684	0.32175	0.50040
C	0.32175	0.00684	0.50040
C	0.50231	0.99769	0.31618
C	0.99769	0.50231	0.31618
C	0.68386	0.49994	0.00003
C	0.00006	0.81614	0.00003
C	0.49994	0.68386	0.00003
C	0.81614	0.00006	0.00003
C	0.50241	0.99759	0.68386
C	0.99759	0.50241	0.68386
C	0.50001	0.50001	0.18446
C	0.99999	0.99999	0.18446
C	0.50005	0.50005	0.81569
C	0.99995	0.99995	0.81569

Table S9-4. The fractional coordinates of the migration path 4 of the optimized structure for Pd-sorbed PB. The lattice constant and angle were set to a, b, c = 10.4841 Å, and α, β, γ = 90º, respectively.

Atom	*x*	*y*	*z*
Pd	0.25000	0.25000	0.67651
Fe	0.50211	0.50211	0.49975
Fe	0.99789	0.99789	0.49975
Fe	0.50054	0.99946	0.00106
Fe	0.99946	0.50054	0.00106
Fe	0.99981	0.99981	0.00074
Fe	0.50019	0.50019	0.00074
Fe	0.99938	0.50062	0.50010
Fe	0.50062	0.99938	0.50010
N	0.29611	0.00050	0.99956
N	0.49950	0.20389	0.99956
N	0.00050	0.29611	0.99956
N	0.20389	0.49950	0.99956
N	0.70347	0.49933	0.50013
N	0.79653	0.00067	0.50013
N	0.00067	0.79653	0.50013
N	0.49933	0.70347	0.50013
N	0.49517	0.29521	0.50447
N	0.29521	0.49517	0.50447
N	0.00483	0.20479	0.50447
N	0.20479	0.00483	0.50447
N	0.49993	0.00007	0.20395
N	0.00007	0.49993	0.20395
N	0.79636	0.49995	0.99998
N	0.00005	0.70364	0.99998
N	0.49995	0.79636	0.99998
N	0.70364	0.00005	0.99998
N	0.49916	0.00084	0.79626
N	0.00084	0.49916	0.79626
N	0.49953	0.49953	0.29699
N	0.00047	0.00047	0.29699

N	0.49928	0.49928	0.70450
N	0.00072	0.00072	0.70450
C	0.18364	0.00002	0.00028
C	0.49998	0.31636	0.00028
C	0.00002	0.18364	0.00028
C	0.31636	0.49998	0.00028
C	0.81598	0.50004	0.49994
C	0.68402	0.99996	0.49994
C	0.99996	0.68402	0.49994
C	0.50004	0.81598	0.49994
C	0.49819	0.18263	0.50223
C	0.18263	0.49819	0.50223
C	0.00181	0.31737	0.50223
C	0.31737	0.00181	0.50223
C	0.50021	0.99979	0.31640
C	0.99979	0.50021	0.31640
C	0.68391	0.50009	0.00033
C	0.99991	0.81609	0.00033
C	0.50009	0.68391	0.00033
C	0.81609	0.99991	0.00033
C	0.50045	0.99955	0.68383
C	0.99955	0.50045	0.68383
C	0.49992	0.49992	0.18448
C	0.00008	0.00008	0.18448
C	0.49981	0.49981	0.81699
C	0.00019	0.00019	0.81699

Table S9-5. The fractional coordinates of the migration path 5 of the optimized structure for Pd-sorbed PB. The lattice constant and angle were set to *a*, *b*, *c* = 10.4841 Å, and *α*, *β*, *γ* = 90°, respectively.

Atom	*x*	*y*	*z*
Pd	0.25000	0.25000	0.75000
Fe	0.50000	0.50000	0.50000
Fe	0.50000	0.00000	0.00000
Fe	0.00000	0.50000	0.00000
Fe	0.00000	0.00000	0.50000
Fe	0.00000	0.00000	0.00000
Fe	0.00000	0.50000	0.50000
Fe	0.50000	0.00000	0.50000
Fe	0.50000	0.50000	0.00000
N	0.29576	0.00000	0.00000
N	0.50000	0.29576	0.50000
N	0.50000	0.20424	0.00000
N	0.29576	0.50000	0.50000
N	0.50000	0.00000	0.79576
N	0.00000	0.50000	0.79576
N	0.50000	0.50000	0.70424
N	0.00000	0.20424	0.50000
N	0.00000	0.29576	0.00000
N	0.20424	0.00000	0.50000
N	0.00000	0.00000	0.70424
N	0.20424	0.50000	0.00000
N	0.70424	0.50000	0.50000
N	0.79576	0.00000	0.50000
N	0.50000	0.00000	0.20424
N	0.00000	0.50000	0.20424
N	0.79576	0.50000	0.00000
N	0.00000	0.70424	0.00000
N	0.50000	0.79576	0.00000
N	0.50000	0.50000	0.29576
N	0.00000	0.79576	0.50000
N	0.00000	0.00000	0.29576

N	0.50000	0.70424	0.50000
N	0.70424	0.00000	0.00000
C	0.18316	0.00000	0.00000
C	0.50000	0.18316	0.50000
C	0.50000	0.31684	0.00000
C	0.18316	0.50000	0.50000
C	0.50000	0.00000	0.68316
C	0.00000	0.50000	0.68316
C	0.50000	0.50000	0.81684
C	0.00000	0.31684	0.50000
C	0.00000	0.18316	0.00000
C	0.31684	0.00000	0.50000
C	0.00000	0.00000	0.81684
C	0.31684	0.50000	0.00000
C	0.81684	0.50000	0.50000
C	0.68316	0.00000	0.50000
C	0.50000	0.00000	0.31684
C	0.00000	0.50000	0.31684
C	0.68316	0.50000	0.00000
C	0.00000	0.81684	0.00000
C	0.50000	0.68316	0.00000
C	0.50000	0.50000	0.18316
C	0.00000	0.68316	0.50000
C	0.00000	0.00000	0.18316
C	0.50000	0.81684	0.50000
C	0.81684	0.00000	0.00000
